# Maximizing the Energy Output of Soft Electrohydraulic Generators

**DOI:** 10.1002/advs.76834

**Published:** 2026-08-07

**Authors:** Sophie Kirkman, Ingemar Schmidt, Lawrence T. Smith, Steven L. Zhang, Shane K. Mitchell, Soo Jin Adrian Koh, Philipp Rothemund, Christoph Keplinger

**Affiliations:** ^1^ Robotic Materials Department Max Planck Institute for Intelligent Systems Stuttgart Germany; ^2^ Paul M. Rady Department of Mechanical Engineering University of Colorado Boulder Boulder USA; ^3^ Institute for Adaptive Mechanical Systems University of Stuttgart Stuttgart Germany; ^4^ Materials Science and Engineering Program University of Colorado Boulder Boulder USA

**Keywords:** electrostatic generators, energy harvesting, HASEL, ocean wave energy, renewable energy, soft transducers, sustainability

## Abstract

Energy harvesting from ocean waves and human motion remains largely unused, despite vast potential. Soft electrostatic transducers are a potential solution, leveraging shape‐dependent capacitance to convert mechanical energy into electricity. While hydraulically‐amplified self‐healing electrostatic (HASEL) transducers are versatile, high‐performance actuators, their potential as generators remains largely unexplored. Through a combined theoretical and experimental approach, this work elucidates how to maximize the energy output of HASEL generators: a quasi‐static analytical model explains the fundamental mechanisms governing the behavior of the generators and enables mapping of their operational limit‐states on work‐conjugate planes; a comprehensive parametric evaluation confirms that the model captures key experimental trends, allowing for the construction of a roadmap toward generators with substantially increased performance. Notably, this work reveals a compressive force causes greater capacitance change and higher electrical energy output than the same force in tension. Guided by the model, the optimized generator design and operating conditions lead to a maximum specific energy and power of 15 J/kg and 43 W/kg, and energy conversion efficiency of 40%, setting new benchmarks in all measured metrics. Built on a foundation of rigorous theoretical and experimental analysis, the roadmap will guide the development of high‐performance electrohydraulic generators for capture of underutilized energies.

## Graphical abstract

Many energy resources cannot be viably harvested with electromagnetic generators. This article evaluates the potential of soft electrohydraulic generators as an alternative approach for energy capture. An analytical model charts their operational limits and a rigorous experimental study results in new performance benchmarks with maximum specific energy of 15 J/kg, specific power of 43 W/kg, and efficiency of 40%.

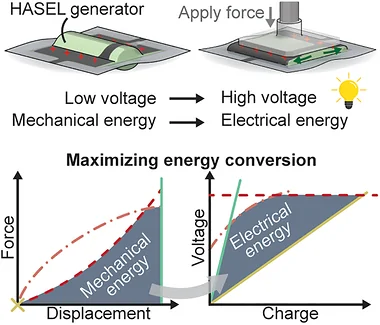

## 1 Introduction

The unused energy all around us—spanning from small‐scale sources such as human and robotic motion all the way to ocean waves—could be used to extend the operation time of portable electronics or mobile robots, and to reduce our reliance on fossil fuels [1, 2, 3]. Remarkably, the electromagnetic (EM) generator can efficiently generate electricity from almost every grid‐scale energy source—from wind to nuclear—because these sources drive rapid, continuous rotations of a turbine (Figure 1A). However, many energy sources are available in the form of slow, intermittent motion, which can only be efficiently coupled to EM generators through complex transmissions. Ocean wave energy is a vast untapped resource, where, along with the low‐frequency oscillatory nature of the waves, the extreme marine environment renders rigid EM generators commercially unviable [2, 3]. There are also smaller‐scale underutilized energy sources, such as energy absorbed from mechanical shocks in human or robotic motion [4, 5]. Electrostatic generators (Figure 1B) show promise in harvesting energy from all these underutilized sources as they can effectively operate at low frequencies [6, 7, 8, 9, 10, 11]. Soft electrostatic generators, such as dielectric elastomer generators (DEGs), leverage large shape change of electroactive materials to harvest energy [12] and their softness allows for impedance matching with waves [8, 9] or for comfortable integration on the human body [13].

**FIGURE 1:**
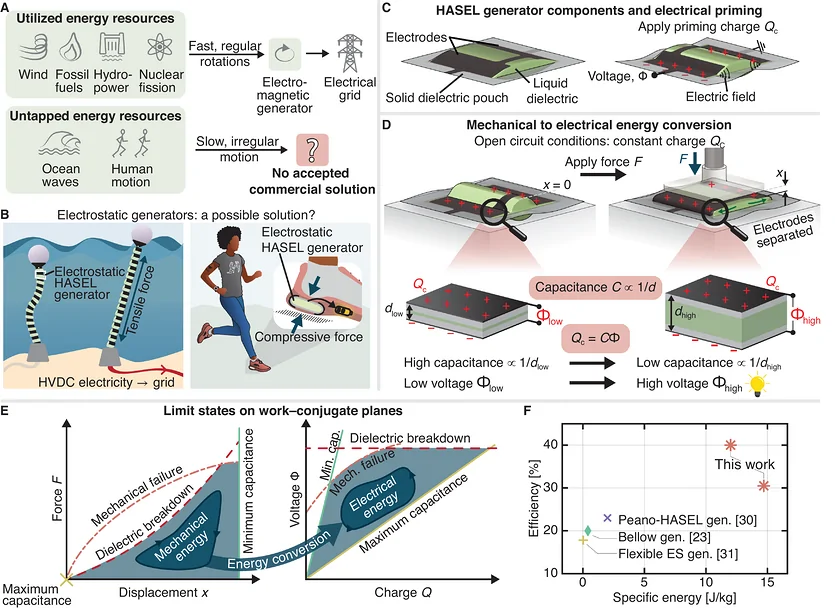
Exploring the maximum energy output of HASEL generators. (A) Most grid‐scale energy sources are harnessed efficiently by the electromagnetic generator. However, there is not yet a viable solution to convert the energy from sources with low‐frequency or irregular motion. (B) HASEL generators could be a solution to harvest energy from currently untapped energy resources. (C) HASEL generators comprise a solid dielectric shell, filled with a liquid dielectric and partially coated with electrodes on both sides. When a voltage is applied across the electrodes they zip together, displacing the liquid between them and causing shape change. (D) A HASEL generator in open circuit conditions has a constant charge *Q*_c_. With no force applied, it is in the maximum capacitance state, with distance *d*_low_ and voltage Φ_low_ between the electrodes. A mechanical force *F* separates the charges against the electric field, which decreases the capacitance of the generator by increasing the distance to *d*_high_, thus forcing the voltage to increase to Φ_high_. (E) We can represent the operational limits of the generator and thus the maximum energy that can be harvested on work‐conjugate planes. (F) HASEL generators outperform all other electrostatic generators which use a liquid dielectric.

While DEGs have shown very high specific energy and power of 780 J/kg [14] and 280 W/kg [15] in laboratory demonstrations, these values may only be realized when the stretchable components (elastomer and electrodes) undergo very large strains, which drastically compromises durability [16]. Operating reliably for many cycles requires a substantial reduction in both operating strains and electric fields, thereby dramatically decreasing the output energy [17]. Additionally, stretchable materials can introduce viscoelastic losses [18]. The potential of DEGs for ocean wave energy harvesting has been studied extensively and despite many years of commercial development [19, 20], there has not yet been any successful large‐scale implementation due to the challenges listed above.

Incorporating fluid dielectrics into electrostatic generators helps to overcome some drawbacks of large deformations of DEGs. Fluid dielectrics deform easily under load [21], reducing the mechanical force required to deform the generator, and they self‐heal, thereby enhancing reliability. Fluids also enable a different mode of capacitance change, electrostatic ‘zipping,’ where two oppositely‐charged electrodes come together progressively and push out the liquid between them. Electrohydraulic transducers have been described by several models [22, 23, 24]. For example, Duranti et al. designed and modeled a generator where a liquid dielectric is pumped into and out of a stretchable pouch to induce zipping and to change capacitance [25]. The same group also developed and modeled a multi‐pouch‐bellow configuration [26], which is deformed by a mechanical force and uses a stretchable reservoir to store the dielectric liquid, achieving a specific energy of 0.41 J/kg and efficiency close to 20%. However, pumping fluids over long distances increases the complexity of energy‐harvesting systems and introduces viscous losses, reducing performance.

Soft electrohydraulic transducers, also known as hydraulically‐amplified self‐healing electrostatic (HASEL) transducers, are typically made of flexible but inextensible sealed pouches in which the dielectric liquid is displaced locally within the pouch as the electrodes zip together, and the liquid serves as a mechanical transmission system (Figure 1C,D). HASEL transducers have high design freedom and do not require large material stretch, thus decreasing mechanical wear and viscoelastic losses, and allowing the use of thin, low‐resistance metal films as electrodes; they can be made from a wide range of materials including low‐cost, commercially available and even fully biodegradable options [27]. HASEL transducers have been successfully used as muscle‐like, high‐performance actuators [28, 29, 30, 31, 32], but only cursorily investigated as generators, achieving specific energies and efficiencies of 2 J/kg and 23% respectively [33, 34]. However, a systematic analysis and understanding of the fundamental performance limits of soft electrohydraulic generators is needed to gauge the potential of this technology across small‐to large‐scale applications.

In this study, we show how to maximize the energy output of soft electrohydraulic generators by developing a quasi‐static model which allows us to map their operational limits on work‐conjugate planes (Figure 1E), and to predict the effect of materials, geometry, and operating conditions and thus to maximize energy output. Our model guided a systematic experimental exploration, which both revealed that compressive loading leads to substantially higher energy output than tensile loading, and set new benchmark performance metrics for this class of generators: specific energy density of 15 J/kg, specific power output of 43 W/kg, and energy conversion efficiency of 40% (Figure 1F) (see Supporting Information for details of other cited studies). Using the predictive capabilities of our model, we offer a strategic roadmap to further improve generator performance through geometry and materials advancements. This work offers a foundation for development of a new generation of soft electrohydraulic generators, thus motivating and enabling the capture of underutilized energies.

## 2 Results

HASEL generators comprise thin dielectric films sealed into a pouch, filled with dielectric liquid, and coated on both sides by electrodes (Figure 1C). When a voltage is applied between the top and bottom electrodes, opposite charges accumulate on them. The resulting electrostatic attraction draws the electrodes together (due to the Maxwell stress), locally displacing the liquid and causing deformation.

To convert mechanical to electrical energy, the generator starts with charge *Q*_c_ at voltage Φ_low_ and the electrodes are zipped together—a high capacitance state (Figure 1D). Under open circuit conditions (charge on the electrodes remains constant), applying a mechanical force will move the liquid to separate the zipped electrodes, thereby reducing the capacitance and increasing the open circuit voltage to Φ_high_ (Φ = *Q*_c_ */C*). The electrical energy of the system is thus increased.

To understand the process of energy conversion in HASEL generators, we plot the evolution of relevant state variables on work‐conjugate force‐displacement (*F–x*) and voltage‐charge (Φ–*Q*) planes (Figure 1E). The area integral of cycles on each plane yields the energy input and output, enabling the evaluation of mechanical‐to‐electrical energy conversion during a closed cycle. We can also plot the bounds that describe the limits of operation on these work‐conjugate planes, thereby allowing us to estimate the maximum possible energy that can be converted [35, 36]. The bounds include the minimum and maximum capacitance of the generator. Dielectric breakdown imposes an upper limit on the electric fields that can be applied to a materials system and the mechanical strength of the HASEL pouch limits the force that can be applied. These limits on the Φ–*Q* and *F–x* planes directly map onto each other when neglecting energy conversion losses, and the area within these bounds provides an estimate of the energy generation potential of HASEL generators. In the next section we derive a model which allows us to understand and maximize this area and thus energy output.

### 2.1 Model for HASEL Generators

We derived a quasi‐static model for a square pouch (Figure 2A,B), using free energy minimization to obtain the equations of state, which we then used to define the operational limits on both the Φ–*Q* and the *F–x* planes (Figure 2C–F). This model also provides insights into the optimal mode of force application, design parameters and operational conditions.

**FIGURE 2:**
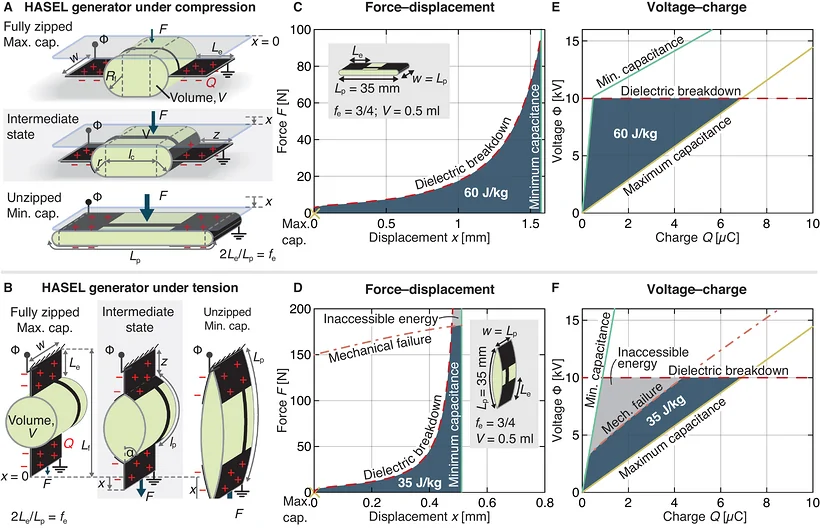
Comparison of compressive and tensile generator modes. (A) In the compressive mode, the cross‐section of the liquid‐filled region is assumed to be rectangular with semicircular sides. (B) In the tensile mode, the cross‐section of the liquid‐filled region is bounded by arc segments. (C, D) The total available energy, represented by area on the force‐displacement planes, is similar for both modes. However, in the tensile mode, the mechanical limit of the film is reached before the generator is unzipped, thus limiting the available energy. (E, F) This difference in available energy is reflected on the voltage‐charge plane: with the same applied force, a generator operated in tension cannot reach the minimum capacitance state at higher voltages, and thus has a lower electrical energy output.

### 2.2 Parametrization of the Geometries

A pouch of width *w* and length *L*_p_ is made of solid dielectric films of thickness *t*_s_ and is filled with liquid dielectric of volume *V*. In this study, the pouch is a square coated with two pairs of connected electrodes, each of length *L*_e_, one pair on the top and the other on the bottom of the pouch (Figures 2A,B and Figure S1) [37]. The electrode coverage *f*_e_ is the fraction of the pouch area that is coated with electrodes ($f_{e}=2\frac{L_{e}}{L_{p}}$). The relative permittivities of the solid and liquid dielectrics are ε_s_ and ε_l_, respectively. We denote the zipped length of each electrode with *z*. Prioritizing a lean and compact model, we neglect the bending stiffness of the films, and we assume that there is no liquid in the zipped region, that the film is inextensible, and that the liquid is incompressible. We neglect the mechanical constraints introduced by the seals at the sides of the pouch.

In the compression mode (Figure 2A), we assume that the pouch is being compressed between two flat rigid bodies. The cross‐section of the liquid‐filled region has a flat top and bottom of length *l*_c_ and semicircular sides of radius *r*, thus forming a discorectangle. The zipped length *z* is the single degree of freedom needed to describe the pouch geometry. The radius *r* is

(1) $$r\left(z\right)=\frac{L_{p}-2z}{\pi }-\frac{1}{\pi }\sqrt{{\left(L_{p}-2z\right)}^{2}-A\pi }$$

where *A* is the cross‐sectional area of the liquid‐filled region of the pouch. The radius of the pouch when the electrodes are fully zipped is *R*_f_ and depends on the volume of the pouch and the electrode fraction (see *Geometry parametrization* in SI Appendix). The displacement *x* of the pouch is defined with reference to the fully zipped state:

(2) $$x=2\left(r\left(z\right)-R_{f}\right)$$

In the tension mode, we assume that the cross‐section of the liquid‐filled region is defined by two intersecting circular arc segments (Figure 2B) [24]. The angle between the segments, 2α, is the single degree of freedom needed to describe this geometry. The arc length of the segments is

(3) $$l_{p}\left(\alpha \right)=\sqrt{\frac{2A\alpha^{2}}{\alpha -\frac{\sin\left(2\alpha \right)}{2}}},$$

The length of the generator when the electrodes are fully zipped, *L*_f_, depends on the volume and electrode fraction (see *Geometry parametrization* in SI Appendix). The zipped length of each electrode is $z=\frac{L_{p}\;-\;l_{p}}{2}\;$. The displacement *x* is defined with reference to the fully zipped state:

(4) $$x=\left(l_{p}\left(\alpha \right)\frac{\sin(\alpha )\;}{\alpha }+2z\right)\;-L_{f}$$

### 2.3 Minimizing the Total Free Energy of the System

The capacitance *C* of the generator comprises the capacitances of the zipped and the liquid‐filled regions of the electrodes. As we assume that the zipped region contains no liquid, the zipped capacitance is determined by the films alone, while the unzipped capacitance is the series capacitance of the films and the liquid (see *Capacitance calculations* in SI, Equations S4 and S7, Figures S2 and S3). We ignore contributions from fringing electric field lines.

The electrical energy stored on a capacitor with charge Q on the electrodes is

(5) $$U_{e}=\frac{1}{2}\frac{Q^{2}}{C\left(y\right)}$$

where *y* represents the degree of freedom, which is α in the tension mode or *z* in the compression mode. Charges are supplied to the HASEL from an ideal voltage source with a constant voltage Φ. When charge flows from the battery onto the generator, the free energy of the battery changes by −ΦQ. A mechanical force *F*, assumed to be constant, acts on the generator doing work Fx(y). The total free energy of the system *U* is

(6) $$U\left(y,Q\right)=\frac{1}{2}\frac{Q^{2}}{C\left(y\right)}-\Phi Q-\mathrm{Fx}\left(y\right)$$

The free energy *U* is first minimized with respect to *Q*, giving the well‐known relation Q=CΦ, which is then substituted back into Equation (7) to give

(7) $$U\;\left(y\right)=-\frac{1}{2}C\left(y\right)\Phi^{2}-Fx\left(y\right).$$

To obtain mechanical equilibrium, we minimize Equation 7 with respect to *y*. Due to the complexity of C(y), the analytical form of $\frac{dU}{dy}=0$ cannot be obtained; hence, we used MATLAB's numerical minimization function *fminbnd*.

### 2.4 Area of Allowable States

We can now use the model to quantitatively analyze the area of allowable states (Figure 2C–F). The maximum capacitance bound corresponds to a HASEL with a prescribed voltage and without an applied mechanical force, thus with the electrodes zipped as far as the incompressible liquid allows, and is determined solely by the geometry and materials of the HASEL (see *Capacitance calculation* in SI). The minimum capacitance bound corresponds to the state with both an applied voltage and mechanical force, in which the electrodes are unzipped. The dielectric strength *E*_bd_ of the materials imposes a maximum voltage Φ_max_ that the materials can withstand before dielectric breakdown: $\Phi_{\max}=2t_{s}E_{\mathrm{bd}}$, where *t*_s_ is the thickness of the film. Depending on the applied electric field, the mechanical force required to overcome the attractive electrostatic force between the electrodes may exceed the mechanical strength of the generator. Consequently, the generator's minimum reachable capacitance in operation may be limited by mechanical failure. Mechanical failure mechanisms include the onset of plastic deformation of the films and rupture of the seals between the films. To calculate the mechanical limits, we assume that the film is a thin shell with a constant tension throughout the pouch. Using the corresponding pouch shape for each force mode (Figure 2A,B), we determine the component of the applied force acting either on the film or the seals, and compare this with the film and seal strengths (see *Mechanical limits* in Supporting Information, Figures S4 and S5).

### 2.5 Comparing the Compression and Tension Modes

To compare the two force modes, we use the same generator design (see *Experimental Section* for the materials and experimental parameters), which implies that the minimum and maximum capacitances and dielectric strength are similar; one would therefore expect a similar electrical energy output. However, due to the different pouch shapes and angles between the electrodes ($\alpha =\frac{\pi }{2}$ for compression and $\alpha <\frac{\pi }{2}$ for tension), a higher mechanical force is required to fully separate the electrodes in the tension mode than in the compression mode. To use all the energy available on the electrical plane, a higher force is needed in the tension mode; a higher force is more likely to exceed the mechanical limit as is the case in the example shown in Figure 2. For this geometry, if the applied force is limited by the material strength or by a particular application and is too low to unzip the HASEL generator in the tension mode, the compression mode will give a larger energy output, as we will experimentally confirm below.

### 2.6 Energy Harvesting Cycle and Setup

In our experiments, the generator is connected between two large charge reservoirs to define a cycle which can be idealized as a rectangular cycle on the Φ–*Q* plane, analogous to a Carnot cycle in thermodynamics (Figure 3A) [38, 39]. This cycle describes a process where the generator pumps charge from a charge reservoir at a lower voltage Φ_L_ to a charge reservoir at a higher voltage Φ_H_, thus increasing the electrical energy of the system. The generator is connected to the charge reservoirs via diodes which direct the flow of charge from the lower to the higher voltage charge reservoir.

**FIGURE 3:**
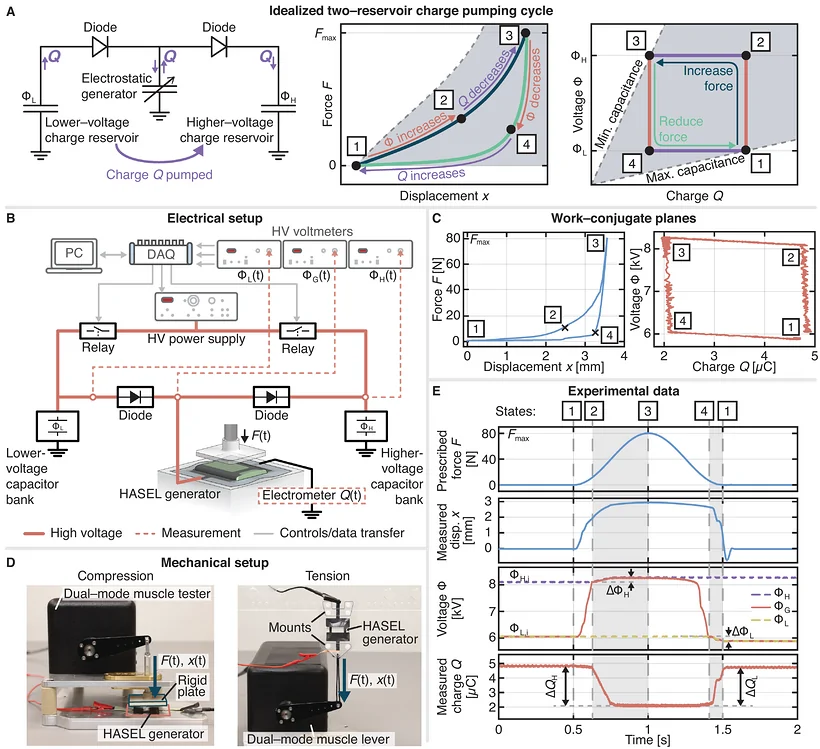
Experimental implementation of two‐reservoir energy harvesting cycle. (A) In the chosen energy harvesting cycle, the HASEL generator pumps charges from a lower‐voltage reservoir to a higher‐voltage reservoir. (B) Two capacitor banks are connected via relays to an HV amplifier and are charged separately to voltages Φ_L,i_ and Φ_H,i_. The generator is connected between the two banks via diodes to force directional flow of charge from lower‐ to higher‐voltage banks. Contactless voltmeters monitor the electrical potential of each capacitor bank and the generator, allowing us to accurately track electrical energy flows. (C) Work‐conjugate planes for one energy harvesting cycle with an applied compressive force of 80 N and with capacitor banks set to initial voltages of Φ_L,i_ = 6 kV and Φ_H,i_ = 8 kV. (D) A dual‐mode muscle lever applies force *F*(t) to the generator and measures the resulting displacement *x*(t). (E) Time‐series data for the cycle in (D).

The cycle starts at state 1, where the generator is connected to the lower‐voltage (L–V) charge reservoir through a diode and thus has a voltage of Φ_L_. With no mechanical force applied, the electrodes are fully zipped and the generator is in the maximum capacitance state. From state 1 to 2, the generator is in an open‐circuit condition because the diode does not allow charge to flow from a higher to a lower voltage. An increasing mechanical force separates the electrodes and decreases the capacitance; the voltage thus increases until it reaches Φ_H_, at which point the diode connects the generator to the higher‐voltage (H–V) reservoir (state 2). From state 2 to 3 the force is further increased and thus continues to decrease the capacitance; as the generator is at a fixed voltage Φ_H_, charge is pumped from the generator to the H–V charge reservoir through the diode. At state 3, the maximum force *F*_max_ and thus the minimum capacitance of the generator is reached. The force is then decreased, and because there are still charges on the generator, the electrodes re‐zip in open‐circuit conditions; the generator's capacitance increases and the voltage thus decreases (states 3–4). At state 4, the generator reconnects with the L–V reservoir through the diode. The force decreases further, allowing the electrodes to continue zipping; the decreasing capacitance at a fixed voltage Φ_L_ causes the generator to draw charges from the L–V reservoir (states 4–1). When the force reaches zero, the generator arrives back at state 1, completing the cycle.

To experimentally implement this cycle (see *Experimental setup and procedure* in SI), we used capacitor banks in place of the idealized infinite charge reservoirs (Figure 3B), where the capacitance of the banks (*C*_bank_ = 15 nF) is much greater than that of the generator (<1 nF). To charge the L–V and H–V capacitor banks to their initial voltages Φ_L,i_ and Φ_H,i_ and to discharge them after experiments, they were connected to a high voltage power supply via relays (Figure S6). Due to finite capacitance in the capacitor banks, the voltages of the banks change as charges Δ*Q*_H_ and Δ*Q*_L_ are pumped to or from them, and we measured these changes with contactless voltage probes. The contactless probes measure the high voltages with minimal interference in the energy harvesting process—it is a very high‐impedance voltage measurement method so minimal charges are lost. In order to visualize the experimental cycle on the Φ–*Q* plane (Figure 3C), we also measured the voltage of the generator and measured its charge using an electrometer on the ground side. While we could use the generator's voltage and charge to calculate the electrical energy output, we rather use the energy stored on the two capacitor banks because it also includes the losses of the capacitor banks and describes the true energy change during one cycle. The electrical energy converted *E*_e_ is therefore calculated by taking the difference of the energy stored in the capacitor banks after and before the cycle:

(8) $$E_{e}=\frac{1}{2}\;C_{\mathrm{bank}}\left(\Phi_{H,f}^{2}+\Phi_{L,f}^{2}-\Phi_{H,i}^{2}-\Phi_{L,i}^{2}\right)$$

where Φ_H,f_ and Φ_L,f_ are the final voltages of the H‐V and L‐V banks. The specific energy is the electrical energy per generator mass (oil and active area of film).

A dual‐mode muscle lever (Figure 3C) applied a sinusoidal force profile to the generator, oscillating between *F* = 0 and *F* = *F*_max_, and measured the resulting displacement *x*, allowing us to visualize the *F*–*x* cycle (Figure 3C). For the compression mode, the generator was compressed between a flat rigid plate and the base plate; in the tension mode, the generator was mounted to an acrylic mount which was connected to the muscle tester via a string. For both cases, the input mechanical work *E*_m_ is given by:

(9) $$E_{m}=\oint F\;dx$$

The efficiency is the ratio of the input to output energy of closed cycles [40]:

(10) $$\eta =\frac{E_{e}}{E_{m}}$$

We ran ten consecutive energy harvesting cycles (Figure 3E), resetting the voltages of the capacitor banks to their initial values between each cycle (Figure S7).

The cycle did not fully close due to charge leakage (Figure 3C). The slope of the trajectory between states 3 and 4 of the Φ–*Q* cycle shows an increase in charge even though it should be in open circuit conditions. In the SI Appendix (*Stray capacitance*, Figure S8), we show that this is likely due to the stray capacitance present in the system.

## 3 Experimental Results and Comparison With Model

For all experimental tests, we used generators made of a polyester film (L0WS MultiPlastics, *t* = 20 µm; ε_s_ = 3.4) and silicone oil (Sigma Aldrich, ε_l_ = 2.6). If not specified otherwise, we prescribe the following: a cycle time of 1 second, the capacitor banks’ initial voltages of Φ_L,i_ = 6 kV and Φ_H,i_ = 8 kV, and a maximum force *F*_max_ of 80 N (Figures 4, 5, 6). We use specific energy as the key metric to validate the model because it gives the energy output of one cycle per unit mass of a generator, which is a useful metric to determine, select, and design light‐weight and low‐cost energy generation systems. The model qualitatively captures all the experimental trends. However, the model consistently overpredicts the specific energy output, which is to be expected as we did not use any fitting factors, we neglected side constraints which reduce the deformation, and we did not consider conversion losses, such as charge leakage. We further report energy conversion efficiency, and studied the dependence of specific energy, power and efficiency on cycle frequency (Figure 6).

**FIGURE 4:**
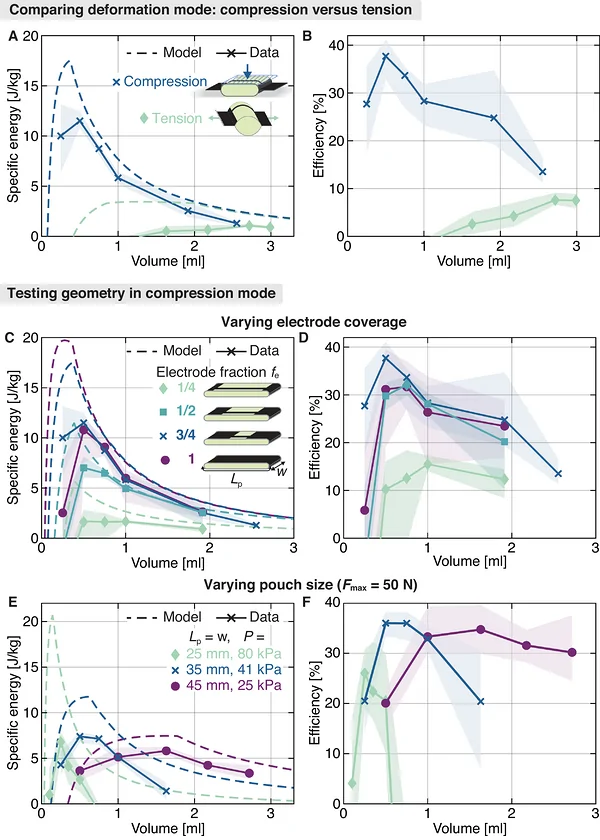
Exploring loading mode and HASEL generator design. Comparing the (A) specific energy and (B) efficiency of generators under compression and tension. As the compression mode produces substantially more energy, it is used in all further experiments. (C) Specific energy and (D) efficiency of generators that have different fractions of the pouch covered by electrodes (*f*_e_). Figures (A‐D) use pouches with length *L_p_* and width *w* of 35 mm, and *F*_max_ of 80 N. (E) Specific energy and (F) efficiency of generators with different pouch sizes, with *F*_max_ of 50 N. All experiments in this figure were operated under the same conditions: Φ_L,i_ = 6 kV and Φ_H,i_ = 8 kV, and cycle duration of 1 second. The data markers show the mean and the shaded area shows the range.

**FIGURE 5:**
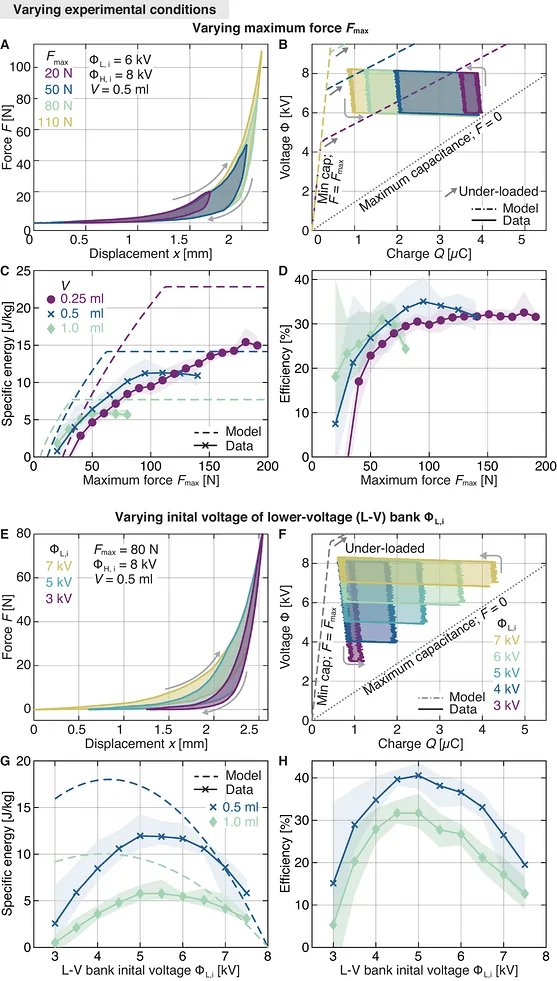
Exploring operational parameters. (A, B) Force‐displacement and voltage‐charge cycles for different applied maximum forces *F*_max_ using capacitor banks with voltages of Φ_L,i_ = 6 kV and Φ_H,i_ = 8 kV. (C) Specific energy and (D) efficiency for varying *F*_max_ and fill volumes. (E, F) Force‐displacement and voltage‐charge cycles for different lower‐voltage bank initial voltages Φ_L,i_, with the higher‐voltage bank initially set to Φ_H,i_ = 8 kV, and with *F*_max_ of 80 N. (G) Specific energy and (H) efficiency for varying lower‐voltage bank initial voltages Φ_L,i_ and fill volumes. All tests were carried out with a generator with electrode coverage *f*_e_ of 3/4 and used a compressive mechanical force. For readability, the cycles were plotted with forces aligned at (A) *F =* 4.5 N and (E) *F* = 80 N, and charges aligned at (B) *Q* = 4 µC and (F) *Q* = 0.6 µC. For C‐D and G‐H, the data markers show the mean and the shaded area shows the range.

**FIGURE 6:**
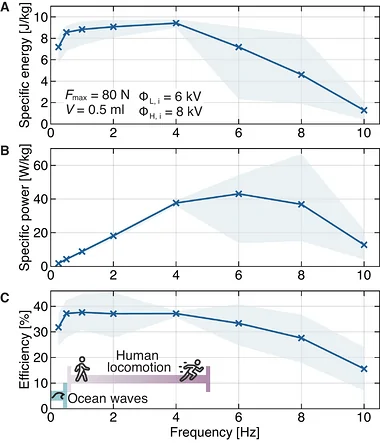
Evaluating energy harvesting performance across frequencies. (A) Specific energy, (B) specific power, and (C) efficiency with respect to frequency. The data markers show the mean and the shaded area shows the range.

### 3.1 Varying Design Parameters

We first confirm the model prediction for our geometry, that for the same force and generator design, the compression mode will give a much higher peak specific energy than the tension mode (Figure 4A). At lower volumes, where the specific energy would be highest, the difference between the two modes is very stark. The specific energy output of the two force modes merges for higher fills, because as the volume increases, a lower force is needed to unzip the electrodes and cause the full capacitance change and thus energy output. The experimental data show a peak specific energy 11 times higher in compression than in tension, so we focus on the compression mode for all further experiments. The efficiency approximately follows the trend of the output specific energy – the higher specific energy output, the higher the efficiency (Figure 4B). We note that some applications might only be compatible with the tension mode of operation, for which the HASEL generator design could be further optimized.

The volume of liquid dielectric is a key design parameter, so we show the performance as a function of volume for all the design parameters. Across all data, there is an optimum volume (assuming a sufficient mechanical force) which results in a peak in the specific energy (Figure 4A,C,E). This is because there are two extreme cases: If the oil volume is zero or if the pouch is completely full, then there is no shape or capacitance change and thus no energy can be harvested. Between these extremes, there is an optimum where the capacitance change and thus energy output is greatest. At the lower and higher volumes, the neglected aspects in the model, such as film‐wrinkling and presence of liquid in the zipped region, exert more influence and so the predictions deviate from the experimental data.

When testing electrode coverage fractions *f*_e_ of ¼, ½, ¾ and 1, the general experimental trend is that the specific energy output increases with increasing *f*_e_ up to a point; the performance of pouches with *f*_e_ = ¾ and *f*_e_ = 1 were very similar, with *f*_e_ = ¾ giving the highest specific energy (Figure 4C). There can be no energy output with no electrodes, *f*_e_ = 0, and according to the model, increasing the electrode coverage should increase the output until the electrodes can fully zip and push the liquid into a cylinder. This means that the higher the volume, the lower the optimum electrode coverage. From the point where the HASEL is fully zipped, the energy output marginally decreases with increasing *f*_e_. At higher electrode coverages, the data do not follow the predicted trends because the electrodes do not follow the assumed zipping pattern. Again, designs with higher electrical energy output displayed a higher efficiency (Figure 4D).

We compared different pouch sizes with pouch lengths and widths of *L*_p_ = *w* = 25 mm, 35 mm, and 45 mm across a range of volumes. When using the same applied force *F*_max_ of 50 N, the peak specific energy is at a higher volume for larger pouch sizes, and the 35‐mm pouch has the highest peak specific energy (Figure 4E). The model predicts that smaller pouches should give higher specific energies; the 25‐mm pouch size did not follow this trend because the wrinkling and bending of the films that make up the pouch become significant at this scale. Furthermore, the model predicts that with the same applied pressure (*F*_max_ /(*L*_p_ *w*)), all pouch sizes have the same peak specific energy, and again the 35‐mm and 45‐mm pouches follow the predictions (Figure S9A,B). As before, the trends in efficiency followed the specific energy output (Figure 4F).

### 3.2 Varying Experimental Conditions

We explored the effect of experimental conditions using the best geometry from above—a 35‐mm pouch with electrode coverage *f*_e_ = ¾ in the compression mode. In this section, we additionally compare the measured and modeled cycles on work‐conjugate planes. The SI Appendix (*Model of F*–*x path*, Figure S10) describes how to calculate the shape of the cycle on the *F*–*x* work conjugate plane.

We studied the effect of the maximum applied force *F*_max_ on the converted energy. A higher force increases energy because it unzips the generator more, leading to a higher deformation (Figure 5A) and decreasing the minimum capacitance (Figure 5B). When *F*_max_ is too low to fully unzip the generator for a given voltage, the minimum capacitance of the generator is not reached (‘under‐loaded’ case in Figure 5B), and the specific energy reduces. Increasing *F*_max_ increases the specific energy until it is high enough to fully unzip the generator, at which point it reaches its geometric minimum capacitance state and the specific energy plateaus (Figure 5C). For higher volumes, the maximum specific energy is lower but is reached at a lower force. The experimental results agree well with the trend predicted by the model for the three tested volumes of 0.25, 0.5 and 1 ml. For the lowest volume, the quantitative agreement is not as good, with the largest deviation at higher forces, where we observed a charge retention effect which caused the electrodes to remain zipped together after discharging, and which substantially reduced performance. We also varied the maximum applied force *F*_max_ for the 45‐mm pouch size, which showed the same trends as the 35‐mm pouch (Figure S9C,D). The efficiency follows the trends shown by the specific energy as before (Figure 5D), except for the lowest volume where the experimental results deviate more from the model.

We then tested the influence of the operating voltage. For the two‐reservoir cycle, the initial voltages of both the lower‐voltage (L–V) and the higher‐voltage (H–V) capacitor bank could be varied independently. However, due to the maximum voltage rating of our components, we kept the H‐V bank's initial voltage Φ_H,i_ fixed at 8 kV. If the voltage difference between the banks is zero or if the voltage difference is too great, no energy can be converted. Varying the initial voltage of the L–V capacitance bank Φ_L,i_ changes the shape of the *F*–*x* and Φ–*Q* cycles (Figure 5E,F), and between the extremes, there is an optimum voltage where the area of the rectangle drawn within the limits on the Φ–*Q* plane is greatest (Figure 5F). While the data follows the predicted shape (Figure 5G), the measured optimum does not agree with the predicted value, which predicts the maximum energy at lower values for Φ_L,i._ The discrepancy could be due to the fact that we neglected any resistance to zipping in the model, meaning that we assume that under no load the generator will fully zip at any voltage (see *Capacitance calculations* in SI for more information). Again, the efficiency follows the trends of the specific energy (Figure 5H).

We also tested the generator performance across a range of frequencies, from 0.25 to 10 Hz. In these experiments, we did two trials of five consecutive cycles (Figure S8). Because our model is quasi‐static, we cannot theoretically predict the effect of frequency. In the frequency ranges of human locomotion (0.5 to 4.5 Hz) [41, 42] and ocean waves (0.05 to 0.5 Hz) [43], the generator exhibited a high specific energy larger than 5 J/kg, reaching a maximum of 8.8 J/kg at 4 Hz. At low frequencies, we attribute the reduced energy output to charge leakage either as corona discharge or through the HASEL and/or capacitor banks (Figure 6A,B). At frequencies above 4 Hz, we observed a drop in specific energy. This drop occurs well before we expect an influence of the viscosity of the liquid dielectric [32]; instead, we observed the same charge retention effect described above. As we increased frequency, specific power increased to 43 W/kg at 6 Hz. The efficiency remained relatively constant up to 8 Hz, reaching a maximum of 37% at 4 Hz (Figure 6C).

### 3.3 Theoretical Roadmap to Increased Energy

Finally, we use the model to illustrate a qualitative picture of how the energy output changes by varying material and geometric parameters using Φ–*Q* curves. As we make simplifying assumptions to create this roadmap, we do not intend to give a quantitative prediction; rather, we show trends and highlight the most important parameters that would substantially change the performance of the generator.

As the reference case (Figure 7A), we use the same generator design and experimental conditions from Figure 2 (listed in *Experimental Section*). Because there is much more energy available than is harvested by our chosen experimental cycle, we consider the entire available area on the Φ–*Q* plane, and we theoretically consider the effect of each parameter on the reference case (Figure 7B–D,F).

**FIGURE 7:**
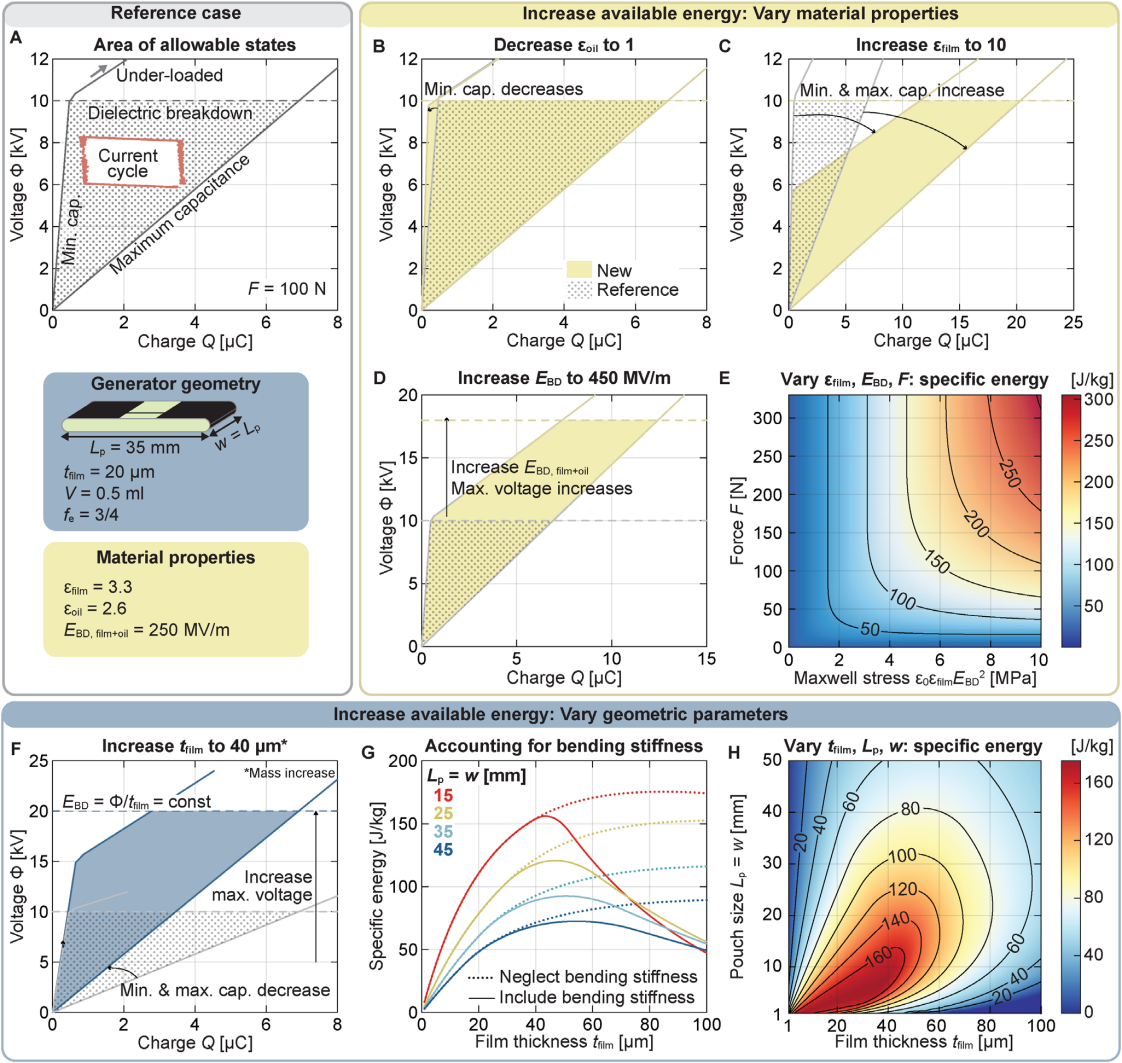
Theoretical roadmap toward generators with substantially increased energy output. (A) Operational limits on the voltage‐charge plane as a reference case to study the effect of varying parameters in isolation (B–D, F). (B) Decreasing the relative permittivity of the oil marginally increases the available energy. (C) Increasing the relative permittivity of the film increases the available energy. (D) Increasing the dielectric strength of the generator increases the maximum operating voltage and available energy. (E) Increasing the applied electric field or the film permittivity both increase the available energy, but only when the mechanical force is large enough to overcome the Maxwell stress (the maximum force of 350 N is from experimental rupture tests for this configuration). (F) Increasing the film thickness while keeping the same maximum electric field increases the operating voltage, leading to higher energy output. In this example, the total mass of the generator increases by 12%. (G) Bending stiffness limits performance when the film thickness increases. (H) Certain combinations of pouch size and film thickness result in drastically increased specific energy output. In this plot, we account for bending stiffness while keeping the same ratio of volume to pouch size and the same electric field.

We first change material parameters: decreasing the oil permittivity decreases the minimum capacitance and has no effect on the maximum capacitance, meaning that the available area increases (Figure 7B). However, the oil permittivity can barely be reduced – a relative permittivity less than 1 (vacuum) is not possible – and so changing the oil permittivity brings only a very modest increase in energy. In contrast, increasing the film permittivity drastically increases both the minimum and maximum capacitance (Figure 7C). While more force is needed to unzip the generator and the ‘under‐loaded’ condition is reached at a lower force, the considerably increased maximum capacitance still leads to a much greater area. It is worth noting that charge retention could occur in some material combinations when the ratios of their permittivity and conductivity differ too much; to prevent this effect, one could change the conductivity of the materials as well [44]. Finally, increasing the dielectric strength (*E*_BD_) of the materials directly increases the available area and thus the energy (Figure 7D).

We next take the two parameters which have the most impact—film permittivity and breakdown field—to show the relationship between the Maxwell stress $\varepsilon_{0}\varepsilon_{\mathrm{film}}E^{2}$ and the mechanical force (Figure 7E). Increasing the field and the film permittivity increases the available area, but the generator will inevitably enter the under‐loaded state, in which the applied mechanical force is not sufficient to overcome the Maxwell stress holding the electrodes together. It is therefore necessary to increase the applied force to profit from the changes in film permittivity and electric field. Again, we neglect material‐dependent effects such as charge retention and we note that simultaneously increasing both the dielectric strength and the permittivity of a dielectric material is an ongoing challenge in materials science, and a practical solution would be to increase only one of these values [45].

Increasing the film thickness *t*_s_ increases the area available on the Φ–*Q* plane because with the same electric field and a thicker film, the voltage is higher and thus the absolute energy is higher (Figure 7F). We assume that the dielectric strength of the film remains constant with thickness [46]. While increasing *t*_s_ somewhat increases the mass (here ∼12%), specific energy still increases with *t*_s_ (Figure 7G). However, the film thickness cannot be arbitrarily increased as the bending stiffness of the film will at some point prevent the generator from zipping. We therefore account for the effect of bending stiffness (see Bending stiffness in SI), and show that the specific energy begins to drop when the ratio of *t*_s_ to pouch length becomes too large (Figure 7G). We further explore the relationship between pouch size and film thickness (Figure 7H). We keep the same electric field and the same pouch size to volume ratio and show that there is an optimum combination of film thickness and pouch size for maximum specific energy. We note that realizing sub‐millimeter generators would be challenging with current fabrication methods. As film thickness decreases, the seal strength will reduce, but the forces on the heat seal reduce too.

## 4 Discussion

This combined theoretical and experimental study provides a holistic understanding of the maximum energy that can be converted by HASEL generators and sets new performance benchmarks for this class of generators with specific power of 43 W/kg, efficiency of 40% and specific energy of 15 J/kg, which is 6 times higher than previous works. For our analysis we chose a simple design consisting of a square pouch with a pair of electrodes printed on each side, for which we revealed that higher energies can be achieved with configurations that require less force to change capacitance, such as operating the generator in compression instead of in tension. Our method and key findings can be generalized to other common HASEL designs; however, to substantially increase specific energy, it would be interesting to broadly explore other electrohydraulic architectures, such as those that feature particularly high capacitance change without increasing the mass.

In this work, we used two capacitor banks and diodes for passive switching, giving a practical and robust energy harvesting cycle which maps a rectangular path on the voltage‐charge plane. However, this rectangular path only captures a fraction of the energy available on the Φ–*Q* plane and a more sophisticated electrical control circuit will increase energy output substantially [47, 48]. Furthermore, overcoming the technical constraints of our experimental setup—a maximum operating voltage of 10 kV and force of 200 N—would unlock higher performance; for ocean‐wave energy harvesting in particular, transporting electricity at higher DC voltages is a benefit as it reduces inductive and resistive losses [49].

While we conducted a broad parametric study to optimize design and experimental parameters of individual HASEL pouches, we used only commercially available and commonly used materials. As well as using higher‐permittivity materials, replacing a portion of the liquid with gas can increase the specific energy by reducing the mass of the generator, and the added buoyancy could be used to orient the generators in the ocean [50]. Here, we focused on individual generators; using arrays could be a promising approach to enable real‐world deployment to harvest energy from ocean waves, as removing and repairing individual, small generators would be easier and cheaper than maintaining large structures [51, 52, 53]. Rigid components, such as the rigid plates in compression and mounts in tension allow for efficient force transmission; however, their added mass would reduce the specific energy of practical devices. We demonstrated that the energy output and efficiency of HASEL generators is largely independent of the frequency of the mechanical stimulus (up to 10 Hz). When considering power density, this feature also opens opportunities to design power takeoff systems which drive multiple energy generation cycles per mechanical input, increasing power density at low frequencies (which could be relevant for ocean waves).

Finally, the presented roadmap will guide further development of soft electrohydraulic generators and inspire the capture of untapped energy sources.

## 5 Experimental Section

For the theoretical comparison of the compression and tension modes (Figure 2) and for the reference case of the roadmap figure (Figure 7), the generator pouch is made of a 20‐µm‐thick film with relative permittivity ε_s_ of 3.4, has an electrode coverage fraction *f*_e_ of ¾ and is filled with 0.5 ml of an oil with relative permittivity ε_l_ of 2.6. We use a force of 100 N and the maximum electric field for which we still get reliable operation in experiment, that is 10 kV or an electric field of 250 MV/m.

For the experiments, the HASEL generators were made of 20‐µm‐thick PET film (relative permittivity of 3.4), silicone oil (relative permittivity of 2.6) and carbon‐based conductive ink and fabricated as outlined in Mitchell et al. [54]. For more information, see *Generator fabrication* in SI Appendix.

For details on the experimental setup, see *Experimental setup and procedure* in the SI Appendix.

## Author contributions

S. Kirkman, P.R. and C.K. conceptualized research, S. Kirkman, S.L.Z., P.R. and C.K. designed experimental setup, S. Kirkman, P.R., L.T.S., and S.J.A. Koh developed theoretical models, S. Kirkman developed software, S. Kirkman, I.S., S.K.M. and P.R conducted experiments, S. Kirkman, I.S. and L.T.S. analyzed data, S. Kirkman and I.S. drafted the initial manuscript and figures, S. Kirkman, I.S., L.T.S., S.L.Z., S.K.M, S.J.A. Koh, P.R., and C.K. edited the manuscript and figures, P.R. and C.K. supervised the research.

## Conflicts of Interest

C.K. and S.K.M. are coinventors on three patents, which cover the fundamentals and basic designs of HASEL transducers (assignee of all three patents is the Regents of the University of Colorado: US Patent 10995779B2, granted 2021‐05‐04; US Patent 11486421B2, granted 2022‐11‐01; and US Patent 11408452B2, granted 2022‐08‐09). C.K. and S.K.M. are cofounders of Artimus Robotics, a start‐up company that is commercializing HASEL transducers. The other authors declare that they have no competing interests.

## Supporting information




**Supporting File**: advs76834‐sup‐0001‐SuppMat.pdf.

## Data Availability

The data that support the findings of this study are available from the corresponding author upon reasonable request.
