# Supplementary material for: Maximizing the Energy Output of Soft Electrohydraulic Generators
Source: Adv Sci (Weinh). 2026 Aug 7;13(59):e76834. doi: 10.1002/advs.76834 (PMC13449534; doi:10.1002/advs.76834)
Supplement: Supplementary file 1 [file ADVS-13-e76834-s001.pdf]

Supporting Information for

Maximizing the Energy Output of Soft Electrohydraulic Generators

Sophie Kirkman[1,2], Ingemar Schmidt[1], Lawrence T. Smith[1], Steven L. Zhang[1], Shane K. Mitchell[2], Soo Jin Adrian Koh[1], Philipp Rothemund[1,2,3]* and Christoph Keplinger[1,2,4]*

[1]Robotic Materials Department, Max Planck Institute for Intelligent Systems, Heisenbergstraße 3, Stuttgart, Germany
[2]Paul M. Rady Department of Mechanical Engineering, University of Colorado Boulder, Boulder, USA
[3]Institute for Adaptive Mechanical Systems, University of Stuttgart, Heisenbergstraße 3, Stuttgart, Germany
[4]Materials Science and Engineering Program, University of Colorado Boulder, Boulder, USA

*Philipp Rothemund: philipp.rothemund@iams.uni-stuttgart.de
*Christoph Keplinger: ck@is.mpg.de

This PDF file contains:

Supporting Information Text

1 Geometry parametrization . . . 2
2 Capacitance calculations . . . 2
3 Mechanical limits . . . 3
4 Bending stiffness . . . 5
5 Generator fabrication . . . 5
6 Experimental setup and procedure . . . 6
7 Stray capacitance . . . 6
8 Model of *F*-*x* path . . . 7

Supporting Information Figures

S1 Photographs of HASEL generators . . . 9
S2 Calculating capacitance in compression mode . . . 10
S3 Calculating capacitance in tension mode . . . 10
S4 Mechanical limits in compression mode . . . 11
S5 Mechanical limits in tension mode . . . 12
S6 Energy harvesting circuit . . . 12
S7 Time series data . . . 13
S8 Stray capacitance in circuit . . . 14
S9 Additional experimental data . . . 15
S10 Model of the F-x and Φ-Q curves . . . 16

Supporting Information Tables

S1 Performance metrics comparison . . . 17

Supporting Information Text

1 Geometry parametrization

The geometry of the pouch when the electrodes are fully zipped depends on the electrode length L_e and the oil volume V.

In the compression mode

The radius of the pouch when the electrodes are fully zipped is R_f. If the electrodes are long enough to push the liquid into a cylinder $(2L_\mathrm{e} \geq L_\mathrm{p} - \sqrt{\frac{V}{\pi w}})$, then:

$$R_\mathrm{f} = \sqrt{\frac{V}{\pi w}}. \tag{S1}$$

Otherwise, we calculate R_f from Eq. 1, with the zipped length equal to the electrode length.

In the tension mode

The generator length when the electrodes are fully zipped is L_f. If the electrodes are long enough to push the liquid into a cylinder ($2L_\mathrm{e} \geq L_\mathrm{p} - \sqrt{\frac{V\pi}{w}}$), then $\alpha = \pi/2$ and

$$L_\mathrm{f} = L_\mathrm{p} - \sqrt{\frac{V\pi}{w}} + 2\sqrt{\frac{V}{\pi w}}. \tag{S2}$$

Otherwise, we solve the expression describing the pouch geometry as a function of α:

$$V = \frac{1}{2}w(L_p - L_e)^2 \frac{\alpha - \sin(\alpha)\cos(\alpha)}{\alpha^2}. \tag{S3}$$

We calculate L_f using Eq. 3 and the following:

$$L_\mathrm{f} = l(\alpha) + 2L_\mathrm{e} \tag{S4}$$

2 Capacitance calculations

When calculating the maximum capacitance of the generators, we assume that the pouch zips at any nonzero voltage without an applied mechanical force. However, in practice, there is some resistance to zipping and a critical electric field is needed to initiate zipping. This means that the maximum capacitance curve has a steeper slope in practice (due to lower capacitance) at low voltages, meaning that there is less energy in the Φ–Q plane when the HASEL is allowed to zip freely. This could be addressed by mechanically pushing the electrodes together during the zipping phase.

Capacitance in the compression mode

The capacitance of the generator in the compression mode consists of the capacitances of zipped and the liquid-filled (unzipped) regions of the pouch. The capacitance of the liquid-filled region (Figures 2A and S2A) comprises the capacitance of the two semicircular regions C_circ and the two flat regions C_flat.

The capacitance of the zipped region is

$$C_\mathsf{zip}(z) = \frac{\varepsilon_0 \varepsilon_\mathrm{s} w}{2t_\mathrm{s}} z \tag{S5}$$

where ε_0 is the permittivity of free space, and ε_s and t_s are the relative permittivity and the thickness of the solid dielectric (film) respectively, w is the width of the pouch and z is the length of the electrode

in the zipped region. If the electrode length L_e is greater than the zipped length z, i.e. $L_\mathrm{e} > z$, we include the capacitance of the circular edges as part of the capacitance of the liquid-filled region. To calculate this quantity, we integrate with respect to β, from the start of the curved portion of the electrode at $\beta = 0$ up to the angular extent of the electrode at $\beta = \bar{\beta}$, where $\bar{\beta} = \frac{L_\mathrm{e}-z}{r(z)}$.

The capacitance of the circular region is therefore:

$$C_\mathrm{circ}(z) = \varepsilon_0 \varepsilon_\mathrm{s} \varepsilon_\mathrm{l} w \int_0^{\bar{\beta}} \frac{r(z)\sin(\beta)}{2t_\mathrm{s}\varepsilon_\mathrm{l} + t_\mathrm{l}(z)\varepsilon_\mathrm{s}}\, d\beta \tag{S6}$$

where ε_l is the relative permittivity of the liquid dielectric and the liquid thickness t_l is: $t_\mathrm{l}(z) = 2r(z)\sin(\beta)$.

One will need to include the capacitance of the unzipped flat section when the electrodes extend past the circular region, so that $\bar{\beta} = \frac{\pi}{2}$ and $L_\mathrm{e} > z + 1/2\pi r(z)$, given as:

$$C_\mathrm{flat}(z) = \frac{\varepsilon_0 \varepsilon_\mathrm{s} \varepsilon_\mathrm{l} w}{2\varepsilon_\mathrm{l} t_\mathrm{s} + \varepsilon_\mathrm{s} r(z)} \left(L_\mathrm{e} - z - \frac{\pi r(z)}{2} \right) \tag{S7}$$

The capacitance of the circular region C_circ barely contributes to the total capacitance of the generators, and can be neglected (Figure S2B), giving the total capacitance of the generator as the sum of (S5) and (S7):

$$C_\mathrm{tot} = 2(C_\mathrm{zip} + C_\mathrm{flat}) \tag{S8}$$

Capacitance in the tension mode

The total capacitance of the generator in the tension mode comprises the capacitances of the two zipped and unzipped regions (Figure S3). We assume that the liquid-filled region of the pouch is defined by two intersecting arc segments with the angle 2α between them (Figure 2B). The capacitance of the zipped region is given by

$$C_\mathrm{zip}(\alpha) = \frac{\varepsilon_0 \varepsilon_\mathrm{s} w}{2t_\mathrm{s}} z(\alpha) \tag{S9}$$

The capacitance of the liquid-filled region is calculated by integrating the series capacitance of the solid-liquid composite along the length of the electrodes that cover the unzipped region of the pouch. The radius r of the circular segment which makes up the oil-filled region is $r(\alpha) = \frac{l_\mathrm{p}}{2\alpha}$ where l_p is the arc length of the unzipped region of the pouch.

$$C_\mathrm{unzip}(\alpha) = \varepsilon_0 \varepsilon_\mathrm{s} \varepsilon_\mathrm{l} w \int_0^{\bar{\theta}} \frac{r(\alpha)\cos(\alpha-\theta)}{2\varepsilon_\mathrm{l} t_\mathrm{s} + \varepsilon_\mathrm{s} t_\mathrm{l}(\alpha)}\, d\theta \tag{S10}$$

where $t_\mathrm{l} = 2r(\alpha)(\cos(\alpha-\theta) - \cos(\alpha))$. We then integrate the unzipped capacitance with respect to θ, from the start of the curved portion of the electrode at $\theta = 0$ up to the angular extent of the electrode at $\bar{\theta} = \theta$, where $\bar{\theta} = \frac{L_\mathrm{e}-z(\alpha)}{r(\alpha)}$.

The total capacitance is therefore the sum of (S9) and (S10):

$$C_\mathrm{tot} = 2(C_\mathrm{zip} + C_\mathrm{unzip}) \tag{S11}$$

3 Mechanical limits

The mechanical limits are governed by the onset of plastic deformation (yield) of the films, rupture of the seals between the films or ripping the mounts off the films. The yield strength σ_yield of the film is an intrinsic material property of the film. The strength of the seal, given as force per length of seal, S_rupture, depends on the sealing method used. In our analysis, we assume that the third failure mode does not govern mechanical failure as it is possible to ensure that the mount is strong enough to avert premature failure. We assume that the seals will only break when the electrodes are fully unzipped. We assume the that the film is a thin shell with constant tension throughout.

Mechanical limits for a generator in compression

Assuming quasistatic deformation, the applied force F produces a hydrostatic pressure P in the pouch acting on the thin film given by:

$$P(z) = \frac{F}{A_\mathrm{c}}(z) = \frac{F}{l_\mathrm{c}(z)w} \tag{S12}$$

where A_c is the contact area of the force, and $l_\mathrm{c}(z) = L - 2z - \pi r(z)$ (Figure S4A). The pressure causes a membrane tension F_H in the film due to hoop stress σ_H, given by:

$$2F_\mathrm{H}(z) = 2\sigma_\mathrm{H} w t_\mathrm{s} = 2Pr(z)w \tag{S13}$$

Using (S12), we get a hoop stress of:

$$\sigma_\mathrm{H}(z) = \frac{r(z)}{wtl_\mathrm{c}(z)} \tag{S14}$$

The film will plastically deform (yield) when the stress in the film is greater than the yield strength of the film σ_yield. This occurs at an applied force $F = F_\mathrm{yield}$

$$F_\mathrm{yield}(z) = wt\sigma_\mathrm{max}\frac{l_c(z)}{r(z)} \tag{S15}$$

We assume that the seal is under the same stress as the film, and the seal will rupture when the force per length exceeds its strength S_rupture. This occurs at an applied force $F = F_\mathrm{rupture}$, which is therefore:

$$F_\mathrm{rupture}(z) = wS_\mathrm{rupture}\frac{l_\mathrm{c}(z)}{r(z)} \tag{S16}$$

Although the heat seal strength of our film is quoted in its data sheet, the strength is dependent on the sealing parameters. To determine the seal strength, we measured the rupture force using a tensile tester (Zwick/Roell Z010 TE) for 35-mm-pouches (Figure S4B). We tested 12 samples for each volume of 0.5 ml and 1.0 ml, and using a Weibull probability distribution gives a rupture force of 330 N and 190 N, respectively.

Fitting (S16) to the measured rupture forces gives a seal strength S_rupture of 60 N/m (Figure S4C). Using this seal strength and a yield strength of σ_yield = 130 $\mathrm{N/mm^2}$ from the L0WS data sheet, we compare the rupture and yield forces with the F–x curve for the reference case from Figure 2A, a 35-mm pouch filled with 5 ml of oil (Figure S4D). The yield force in this example is substantially higher than the force that the generator can produce (Figure 2C), meaning that the film will not deform.

Mechanical limits for a generator under tension

There is constant tensile force F_T and hence stress σ_T acting on the film throughout the pouch (Figure S5), caused by the applied force F:

$$F_\mathrm{T} = \sigma_\mathrm{T} w t_\mathrm{s} = \frac{F}{2\cos(\alpha)} \tag{S17}$$

The film will yield when the stress in the film is greater than the yield strength of the film σ_yield. This occurs at an applied force $F = F_\mathrm{yield}$, from (S17):

$$F_\mathrm{yield}(\alpha) = 2\sigma_\mathrm{yield} wt\cos(\alpha) \tag{S18}$$

The horizontal component of the force F_T will act on the seals of seals with force F_seal and force per unit length of S_seal:

$$F_\mathrm{seal}(\alpha) = S_\mathrm{seal} w = F_\mathrm{T}\sin(\alpha) \tag{S19}$$

We use (S17) to get:

$$F_{\text{seal}}(\alpha) = F\frac{\sin(\alpha)}{2\cos(\alpha)} = F\frac{1}{2\tan(\alpha)} \tag{S20}$$

The seal will break when the force on the seal exceeds its strength: $F_{\text{seal}} \geq S_{\text{rupture}}w$. Hence, the applied force that causes rupture is given by:

$$F_{\text{rupture}}(\alpha) = \frac{2S_{\text{rupture}}w}{\tan(\alpha)} \tag{S21}$$

Again, mechanical failure will occur when the smaller of the two forces F_{rupture} and F_{yield} is reached. In the reference case shown in Figure 2D, the film will yield before the pouch is fully unzipped. Because we only use this geometry for the experiments to compare the compression and tension modes (Figure 4A), we do not experimentally validate the mechanical tests.

4 Bending stiffness

When the film thickness is increased or the pouch length is decreased, as in Figure 7, the bending stiffness of the film can no longer be neglected. In Figures 7G and 7H, we therefore include the bending stiffness of the film, following the method in [S4]. For a rough estimate of the bending stiffness in the compression mode, we assume the shape shown in Figure 2A, neglect side constraints and only consider the bending stiffness of the material in the active area of the pouch. We further assume that bending strain in the film is small enough that it can be modeled as linear elastic.

The contribution of the bending energy to the free energy in Main Text Equation 7 is given by

$$U_{\text{b}} = \frac{1}{2}D\kappa(r)^2 l_{\text{b}}(r) \tag{S22}$$

The curvature κ is given by $\kappa = 1/r$ and the length of film l_{b} to $l_{\text{b}} = 2\pi r$. The bending stiffness of the film D is estimated using the Kirchhoff-Love plate theory [S9]:

$$D = \frac{t^3 wE}{12(1-\nu^2)} \tag{S23}$$

The Young's modulus E and the Poisson's ratio ν are assumed to be 200 MPa and 0.33, respectively. [S1]

5 Generator fabrication

Square HASEL generator pouches were fabricated by heat-sealing together two 20-μm polyester films (L0WS Multi-Plastics) with a CNC (Computer Numerical Control) heat-sealer as demonstrated by Mitchell et al. [S5] To account for the width of the heat seal, the design of the pouch was always 1 mm wider and longer than the electrode pattern. For simplicity, we name the size of the generators by the length and width of the electrode pattern. Two connected electrodes were screen-printed on the top and bottom of each side of the pouch, covering the entire width of the pouch [S6] using carbon-based ink (CI-2051 Nagase-Chemtex).

Generators of lengths and widths 35 mm had a range of electrode sizes to partially cover the length of the pouch by the fractions of the pouch length: ¼, ½, ¾, and 1 (Figure S1). We made two further different pouch sizes, with pouch length L_p = width w of 25 mm and 45 mm, and both of these pouch sizes had ¾ of the pouch area covered by electrodes.

All generators were filled with 5 cSt silicone oil (Sigma-Aldrich). For each size, we used a range of volumes, which we measured and dispensed using a syringe. For the generators to be operated under tension, we bonded acrylic mounts to the top and bottom of the pouches using a few drops of chloroform ($\mathrm{CHCl_3}$).

6 Experimental setup and procedure

Each of the two 15 nF HV capacitor banks consist of a 10 nF (HVP TPM150-10) and a 5 nF (HVP TPM150-102) capacitor. A high voltage amplifier (Trek 610E) is connected and disconnected to the capacitor banks through HV reed relays (Meder HM12-1A69-150). To prevent voltage spikes when opening and closing the relays, a resistor (100 kΩ) is placed in series with the HV amplifier (Figure S6). The relays are switched with a 5V signal to an N-channel MOSFET (IRF540N). HV diodes (VMI SMF-150) allow charge to flow between the the generator and the capacitor banks. Contactless HV probes (Trek 341B) measure the voltages of the banks and the generator and an electrometer (Keithley 6514) measures the charges Q_G on the generator. A dual-lever muscle tester system (Aurora Scientific: 310-LR) applies the mechanical force to the generator and measure the resulting displacement. For HASELs under tension, a Kevlar string connected the MTS with the mount of the generator. For HASELs under compression, a flat rigid plate was used to compress the generator.

The output signals to the amplifier, the muscle tester system (force) and the relays, and the input signals from the voltmeters and muscle tester (displacement) are sent and received by a data acquisition system (NI CDAQ-9174, 9215, 9264). A custom MATLAB program is used to create the output signals and to save the input signals.

When collecting data, for each sample we execute ten consecutive energy harvesting cycles, with each cycle lasting one second. Between each cycle, the voltages of the capacitor banks are reset to their initial values (Figure S7). Since the performance of first few cycles differs from the following cycles, the last six cycles are used to calculate the mean and range. When testing the geometry (Figure 4), we measured three samples. When testing operational conditions (Figure 5), we used two samples and measured the full range for each sample; we tested one sample with increasing force or voltage and the other in decreasing order.

When measuring the effect of frequency (Figure 6), we ran 2 sets of 5 continuous cycles and used the results of the second set. We used four samples for the full frequency range. Due to charge accumulation at higher frequencies, we measured from low to high frequency for each samples.

There were three occasions where a small electric arc discharged the high-voltage capacitor bank–these cycles were omitted from the results.

7 Stray capacitance

This section attempts to explain the portion of the Φ-Q cycle where charge appears to increase (state 3 – state 4) although the generator should be in open circuit conditions (Figure S8A). The generator should also be in open circuit from state 1 to 2, and here we observe that charge decreases (but this could also be due to charge leakage). We posit that charges are stored in the system through the stray capacitance between components at different potentials, and that these charges move around as the voltages of the capacitor banks change.

We assume that there is a 'stray' capacitor in parallel with the generator, and model it by including an extra capacitor in the circuit (Figure S8B). By conservation of charge, the sum of charges initially stored on the generator $Q_\mathrm{g,i}$ and the stray capacitor $C_\mathrm{s}\Phi_\mathrm{i}$ at state 3 (or state 1) of the cycle is equal to the sum of the charges stored on the generator Q_g and on the stray capacitor $C_\mathrm{s}\Phi$ at any later point between state 3 and state 4 (or between states 1 and 2):

$$Q_\mathrm{g,i} + C_\mathrm{s}\Phi_\mathrm{i} = Q_\mathrm{g} + C_\mathrm{s}\Phi \tag{S24}$$

Rearranging (S24) we see that for the same change in voltage, the greater the stray capacitance, the greater the change in charge:

$$C_\mathrm{s} = \frac{Q_\mathrm{g,i} - Q_\mathrm{g}}{\Phi - \Phi_\mathrm{i}} \tag{S25}$$

To test this theory, we added extra 'stray' capacitors to our experimental setup with a value of 70 pF or 160 pF (Figures S8 C,D). As the size of the added 'stray' capacitor increases, the change in

charge also increases, suggesting that stray capacitance is a likely cause of charge increase in open circuit conditions.

8 Model of *F-x* path

The model developed in the results section assumes constant voltage and allows us to predict the electrical energy output of a cycle because our cycle describes a rectangular trajectory in the Φ-*Q* plane, whose area is fully defined by the position of the corners corresponding to the lowest and highest force states at defined voltages (states 1 and 3, marked in Figure 3A).

As well as predicting the electrical energy output, we wanted to additionally validate the model by comparing the measured (Figure 5A,E) and predicted *F–x* trajectories. Because the cycle consists of sections of constant charge and of constant voltage, this requires two additional steps: the formulation of an equation $U(z, V)_{\mathrm{Q}}$ describing the total free energy of the system under constant-charge conditions, and implementation of logic that determines when to transition between constant-charge and constant-voltage conditions. The electrical energy stored on a capacitor with charge Q is

$$U(z)_{\mathrm{e}} = \frac{1}{2}\frac{Q^2}{C(z)} \tag{S26}$$

where z represents the translational degree of freedom in the mechanical domain. Under constant-charge conditions, the generator is electrically isolated from any voltage sources, so the total free energy of the system U_Q is simply

$$U(z)_{\mathrm{Q}} = \frac{1}{2}\frac{Q^2}{C(z)} + Fy(z) \tag{S27}$$

We determine the lowest energy state by minimizing $U(z)_{\mathrm{Q}}$ using MATLAB's general purpose univariate nonlinear minimization function *fminbnd*.

In order to determine when the system transitions between the constant-voltage condition governed by Eq. (8) and the constant-charge condition governed by (S27), we identify the equilibrium force at the corners of the rectangular cycle marked by 2 and 4 in Figure 3A. These forces are determined sequentially, starting on the vertical constant-charge line that begins at State 1 and solving for the force at State 2. As the value of Q and Φ are known at State 2, the capacitance C_2 can be found using $Q_{\mathrm{H}} = C_2\Phi_{\mathrm{H}}$. For any given force F up to the maximum available force, we can use numerical minimization of (S27) to determine the value of the generator's capacitance C at static equilibrium. We define an error function that quantifies the difference between C and C_2 as

$$e_{\mathrm{C}} = (C - C_2)^2 \tag{S28}$$

and use a second call to *fminbnd* to drive this error to a minimum, yielding the force F_2 that satisfies equilibrium and drives the system capacitance to the known target value C_2. Once this force is known, we define a vector of intermediate force values between 0, the force at State 1, and F_2, the force at State 2, and numerically minimize (S28), the free energy in a constant-charge system for each intermediate force.

As the force at State 3 is the pre-defined maximum force $F_{\max}$, we define a vector of intermediate forces between F_2 and $F_{\max}$ and numerically minimize Eq. (8), the free energy in a constant-voltage system for each intermediate force.

Solving for the force at State 4 is the same process for F_2 described above, because the capacitance at this state C_4 can be determined using the relation $Q_{\mathrm{L}} = C_4\Phi_{\mathrm{L}}$. Finally, we solve for equilibrium values of the state variables using a constant charge assumption for the process between states 3 and 4, and a constant voltage assumption for the process between states 4 and 1.

If the force required to reach state 2 exceeds the maximum available force, we terminate the model immediately, as this condition corresponds to a zero-area loop in the Φ-*Q* plane; no charges are pumped until the system reaches the voltage of the upper capacitor bank Φ_{hi}.

When comparing to the experimental data from Figure 5, the model predicts similar trajectories in both the Φ-*Q* and *F-x* planes as are observed in experiments. Notably, with no dissipation or loss

terms included in the model, it predicts 100% efficient conversion between mechanical and electrical energy. This explains why the area included in simulated *F-x* trajectories is uniformly smaller than measured in our experiments on HASEL generators, which convert mechanical to electrical energy with an efficiency between 20 and 40%, depending on geometry and loading conditions.

When compared to experimental data with varying maximum applied force, the model accurately predicts the increase in pumped charges between states 2 and 3, which is represented by the widening of the rectangular loop in the Φ-Q plane (Figure S10 A,B). When compared to experimental data with varying L-V bank voltage, the model predicts the decrease in cycle energy when the difference in bank voltage is either too small or too large (Figure S10 C,D).

Supporting Information Figures and Tables

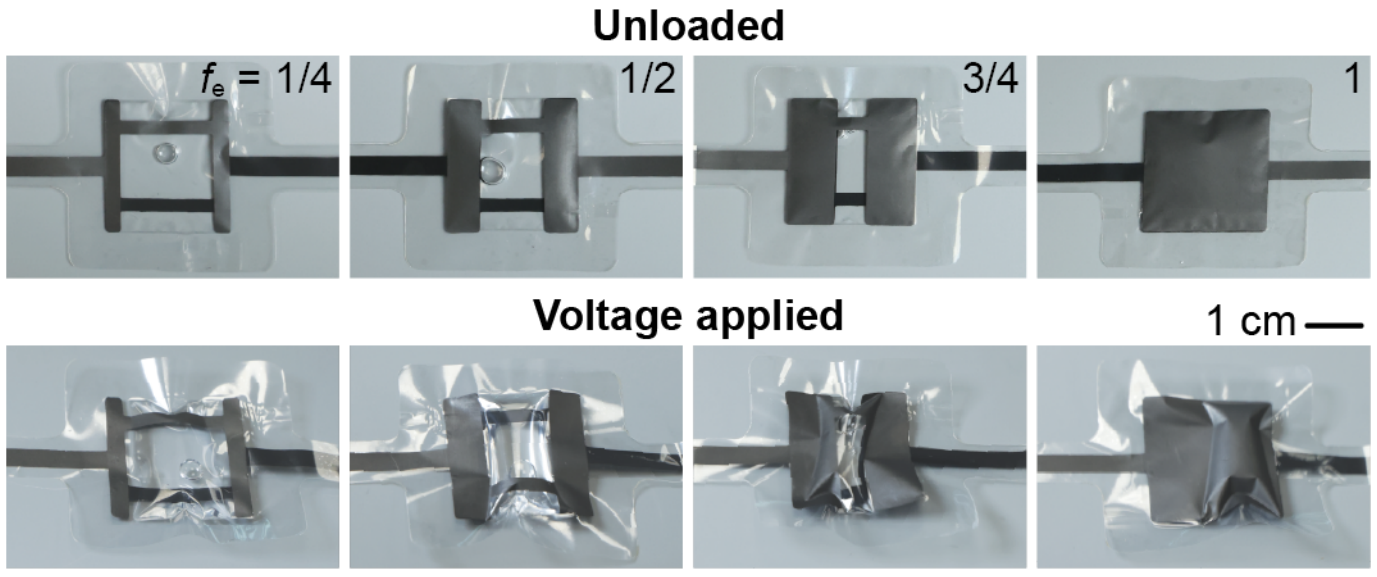

Figure S1. Photographs of 35-mm HASEL pouches made of PET film, filled with 1.5 ml of silicone oil, and coated with different electrode coverage fractions f_e.

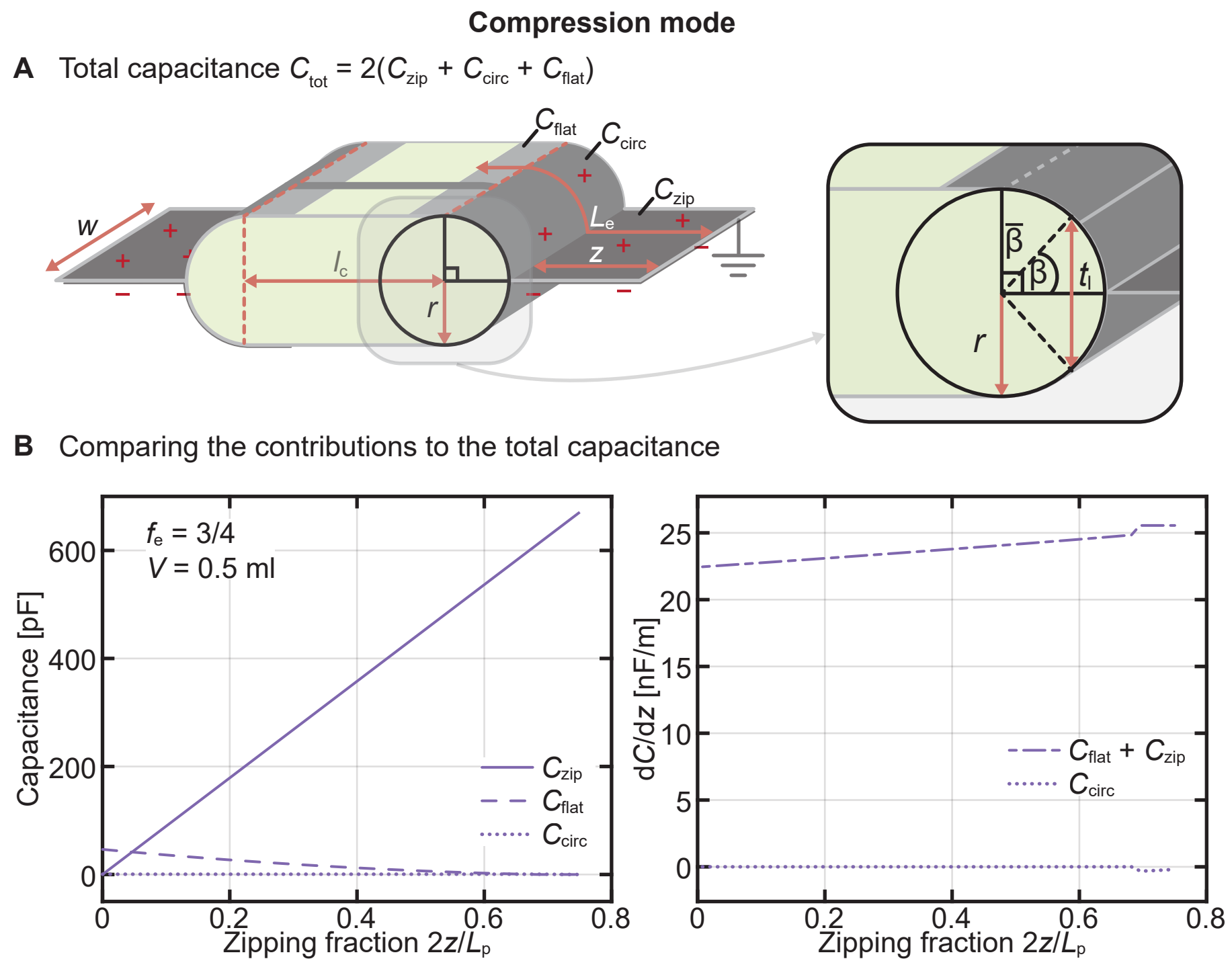

Figure S2. Calculating capacitance in compression mode. (A) In the compression mode, the total capacitance is comprised of three components, C_{zip}, C_{flat} and C_{circ}. If $C_{\text{flat}} > 0$, the electrodes extend past the circular portion and onto the flat portion. (B) The contributions to capacitance of the different regions of the generator, using the generator configuration and experimental conditions from Figure 2, confirming that it is reasonable to neglect C_{circ}.

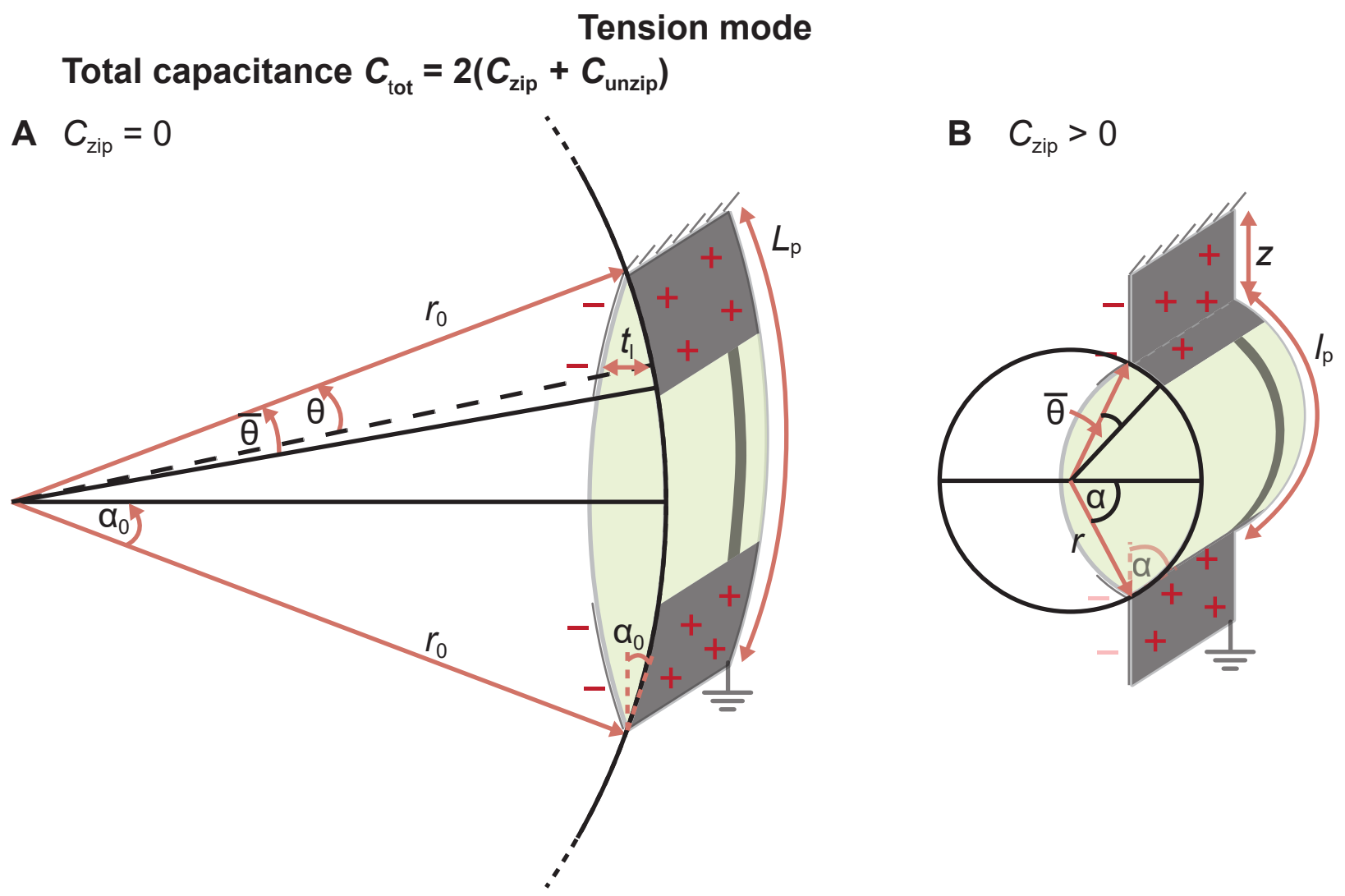

Figure S3. Calculating capacitance in tension mode. In the tension mode, the capacitance consists of the zipped and unzipped capacitances, C_{zip}, and C_{unzip}. (A) The zipped capacitance is zero, $C_{\text{zip}} = 0$. (B) The zipped capacitance is nonzero, $C_{\text{zip}} > 0$.

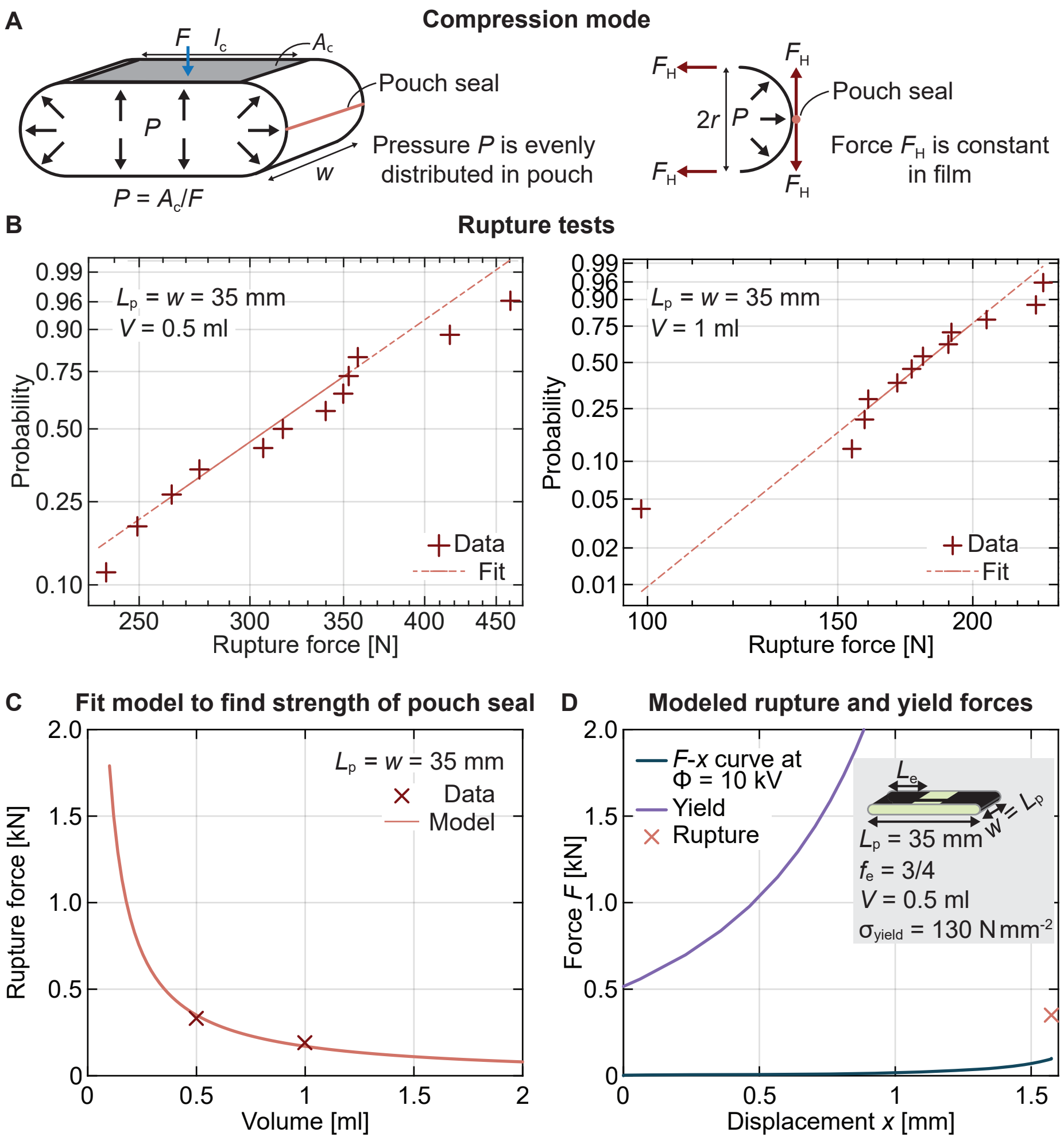

Figure S4. Evaluating the mechanical limits in the compression mode. (A) Pressure is evenly distributed in the pouch and the tension in the film is equal all the way around the pouch. The force due to the hoop stress F_{H} will either cause the seals to pop or the film to plastically deform. The pressure in the pouch is applied force F per contact area A_{c}. (B) Experimentally measured rupture tests for 35-mm-pouches, with 12 samples for each volume of 0.5 ml and 1 ml, fitted to a Weibull distribution, giving rupture forces of 330 N and 190 N, respectively. (C) Fitting the modeled rupture force to the data from (B) gives a seal strength of 60 N/mm. (D) The modeled rupture force, yield force and *F*-*x* curve for a generator under compression with 0.5 ml of liquid dielectric and under 10 kV. The force needed to unzip the generator is much below the yield force. We assume that the pouch will only rupture when it is fully unzipped, and this is plotted as a point. The yield strength of the film is taken from the L0WS data sheet: σ_{yield} is 130 $\mathrm{N/mm^2}$.

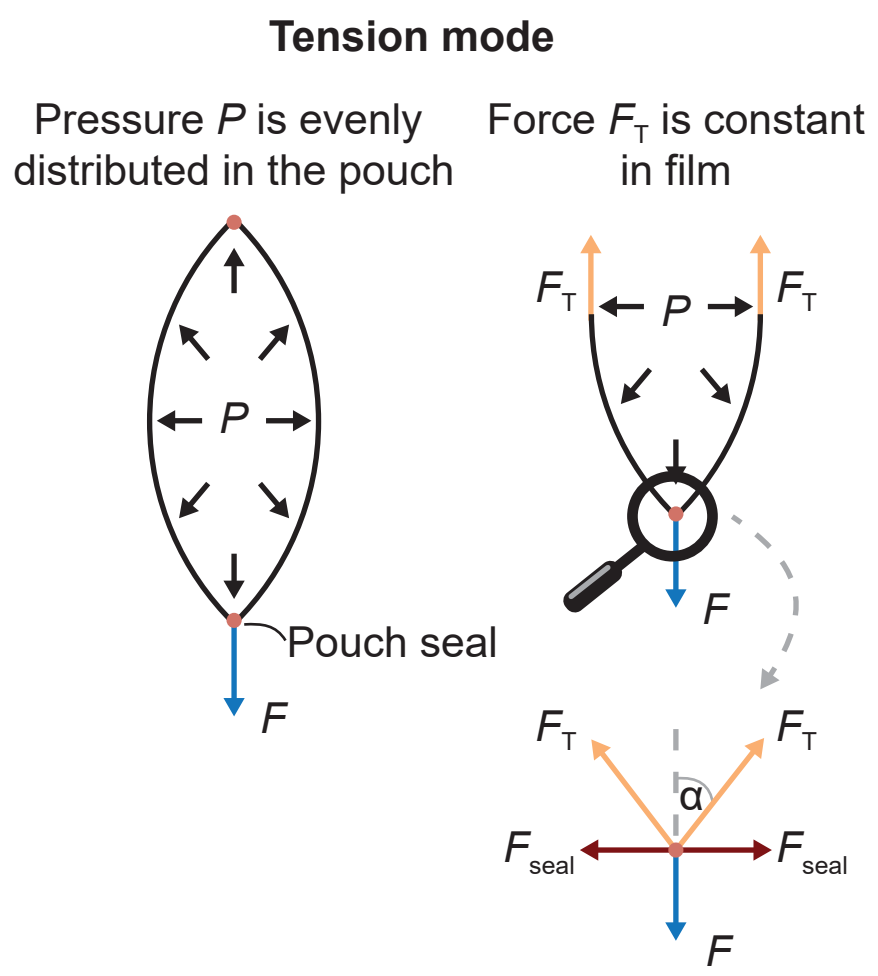

Figure S5. Calculating the mechanical limits in the tension mode. Pressure is evenly distributed in the pouch and the tension in the film is equal all the way around the pouch (circular assumption). The vertical component F_{T} of the applied force causes plastic deformation, while the horizontal component F_{seal} will cause the seal to break.

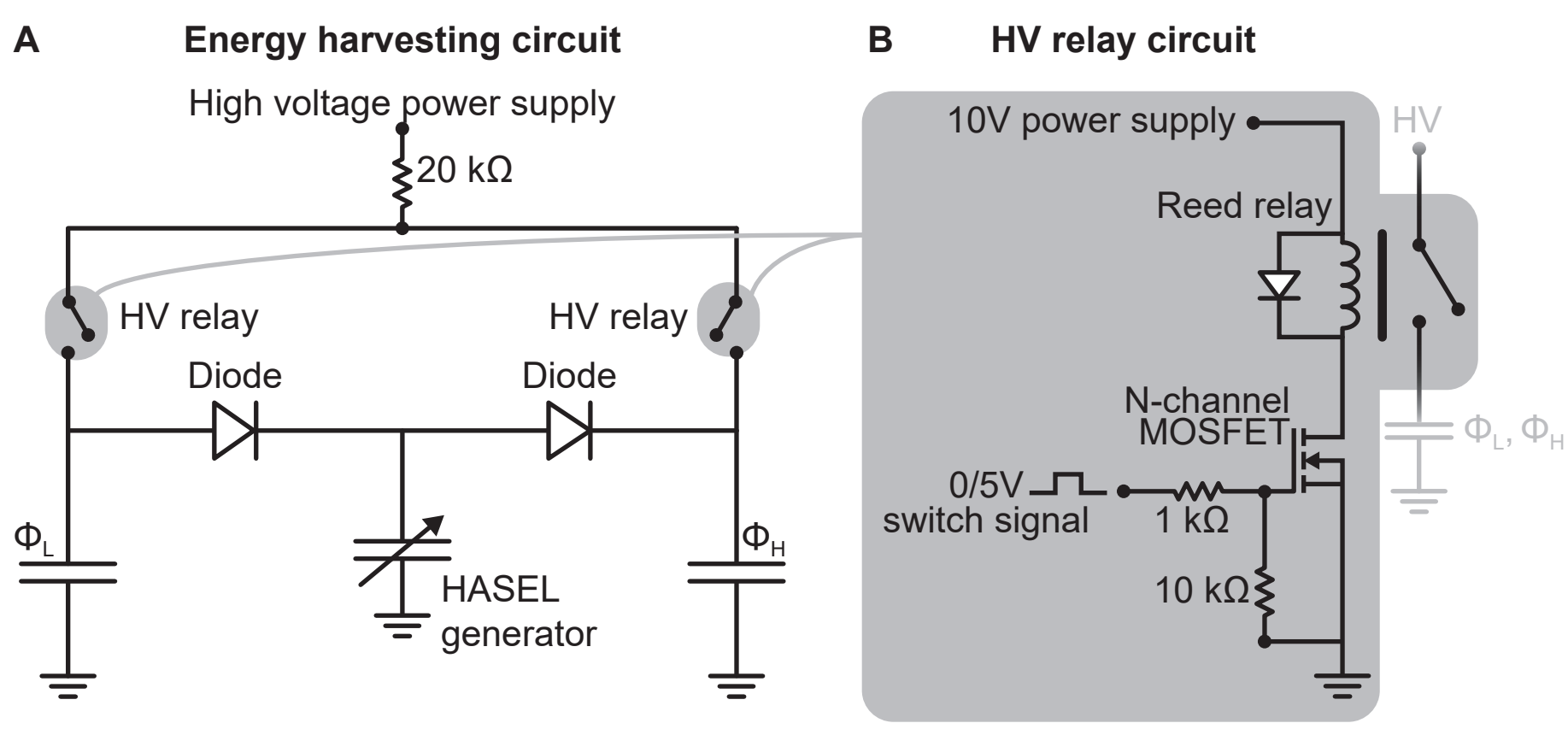

Figure S6. Energy harvesting circuit. (A) Circuit to connect and disconnect the high voltage supply to the lower-voltage and higher-voltage capacitor banks. (B) The circuit to open and close the relays with a 0/5 V signal.

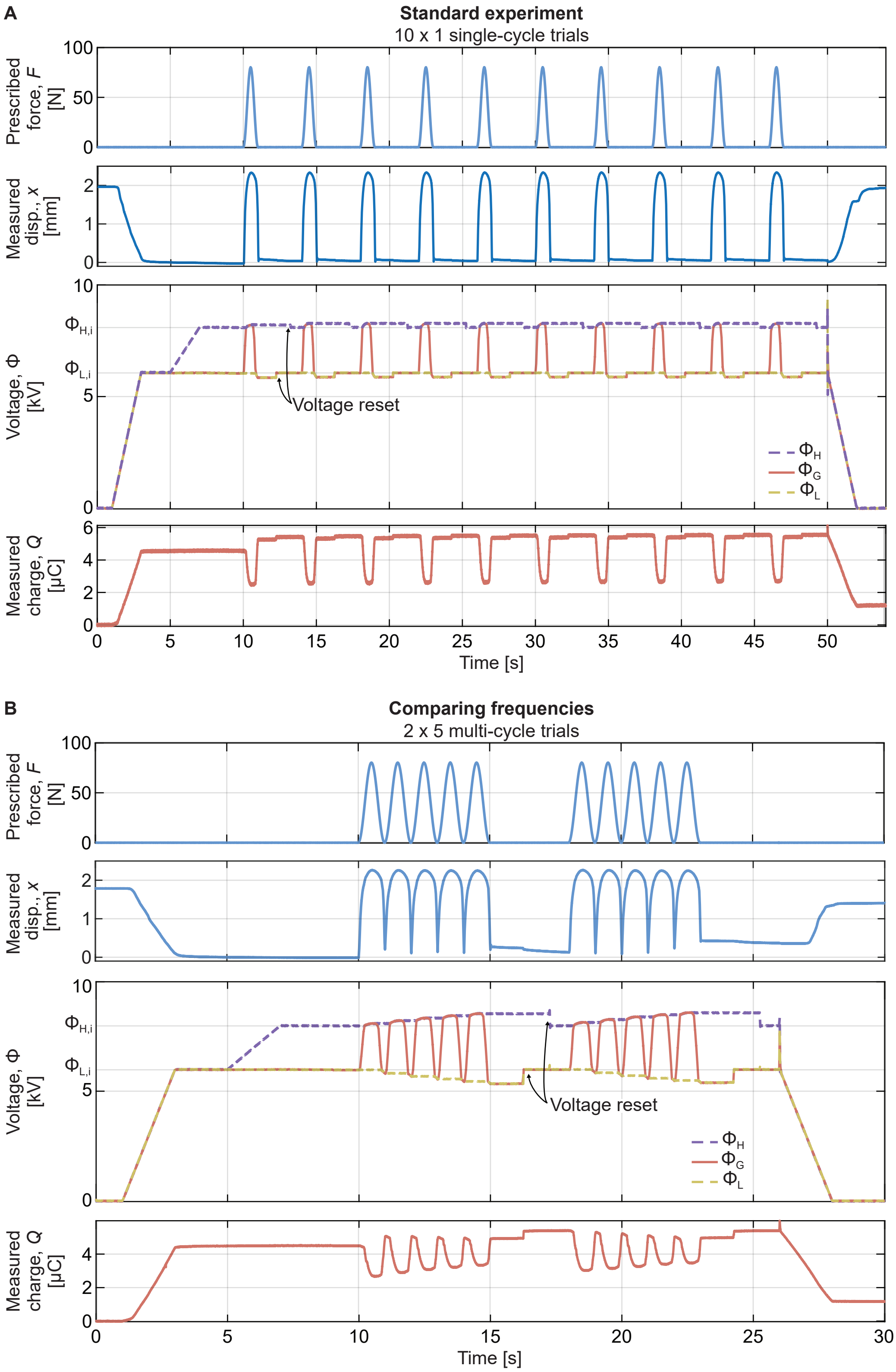

Figure S7. Time series data. (A) A series of ten single cycles (10 x 1) was used for all experiments shown in Figures 4 and 5. (B) The experiments testing the effect of frequency (Figure 6) used two sets of five cycles (2 x 5), where the second set was used for performance calculations.

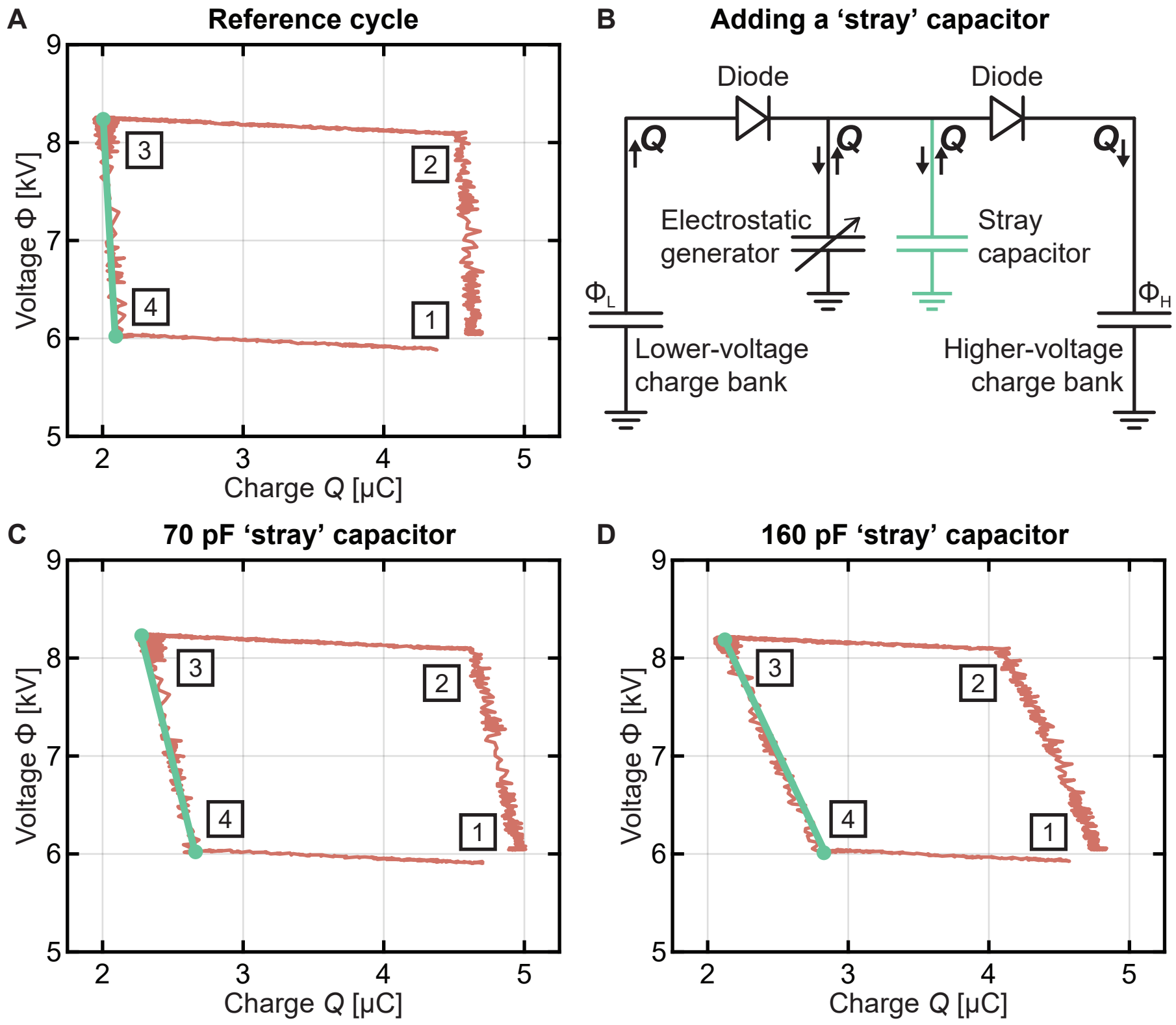

Figure S8. Testing the hypothesis that the slope in the open-circuit conditions is due to stray capacitance. (A) The reference Φ–Q curve from Figure 3C, highlighting the slope between stages 3 and 4. (B) Placement of the extra capacitor that represents the stray capacitance of the system. Adding a 'stray' capacitor with a capacitance of (C) 70 pF or (D) 160 pF proportionally increases the slope between states 3 and 4 (highlighted in green), confirming our hypothesis.

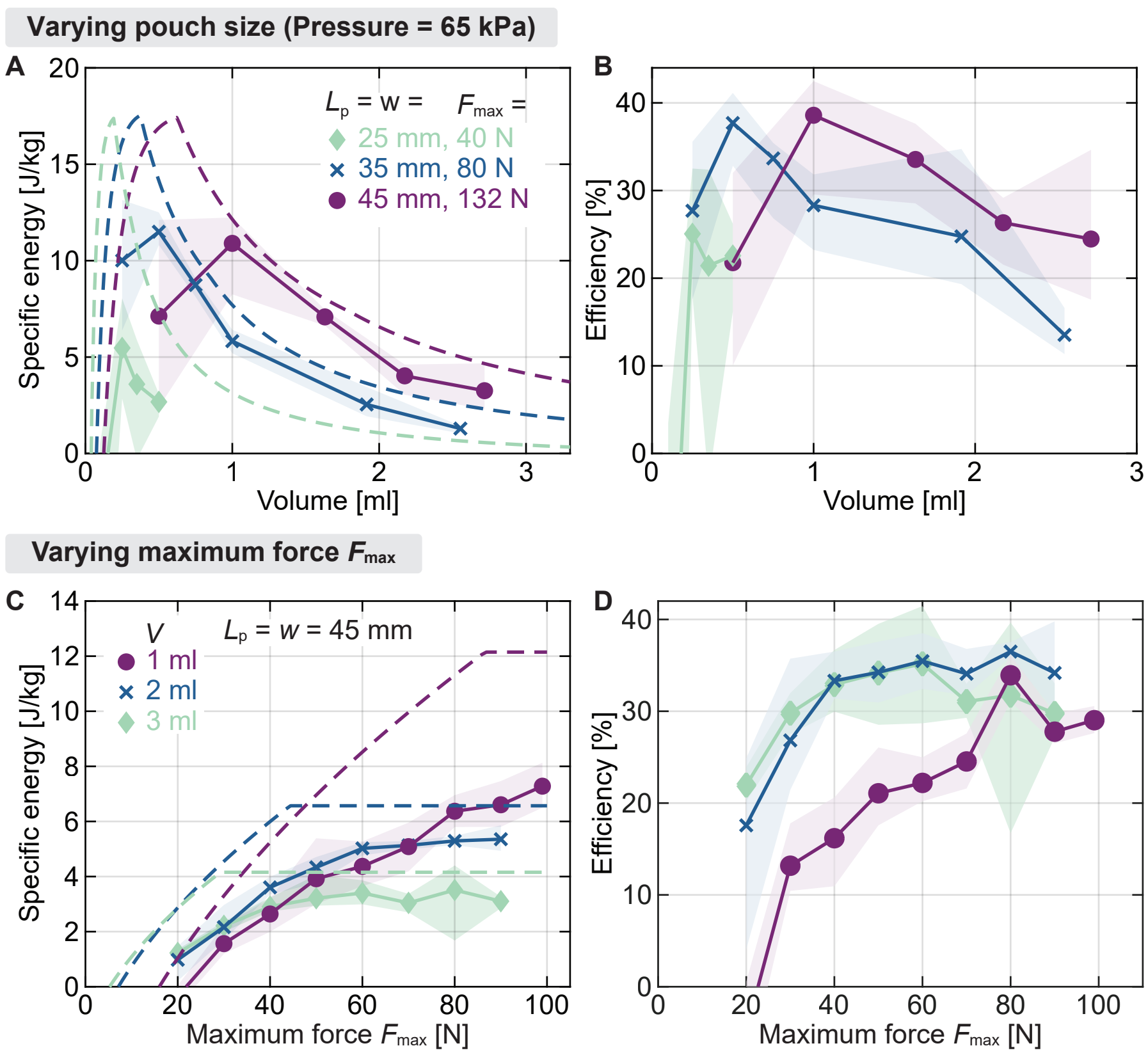

Figure S9. Additional experimental data. (A) Specific energy and (B) efficiency of 3 different pouch sizes, as in Figure 4E, but here using the same pressure of 65 kPa (and hence, different maximum forces $F_{\max}$). (C) Specific energy and (D) efficiency for varying applied maximum forces $F_{\max}$, as in Figure 5 (A-D), but here using a larger pouch size of 45 mm. For all experiments, the cycle duration was 1 second, the capacitor banks' initial voltages were 6 kV and 8 kV, and all pouches had an electrode coverage of 3/4. The data markers show the mean and the shaded area shows the range.

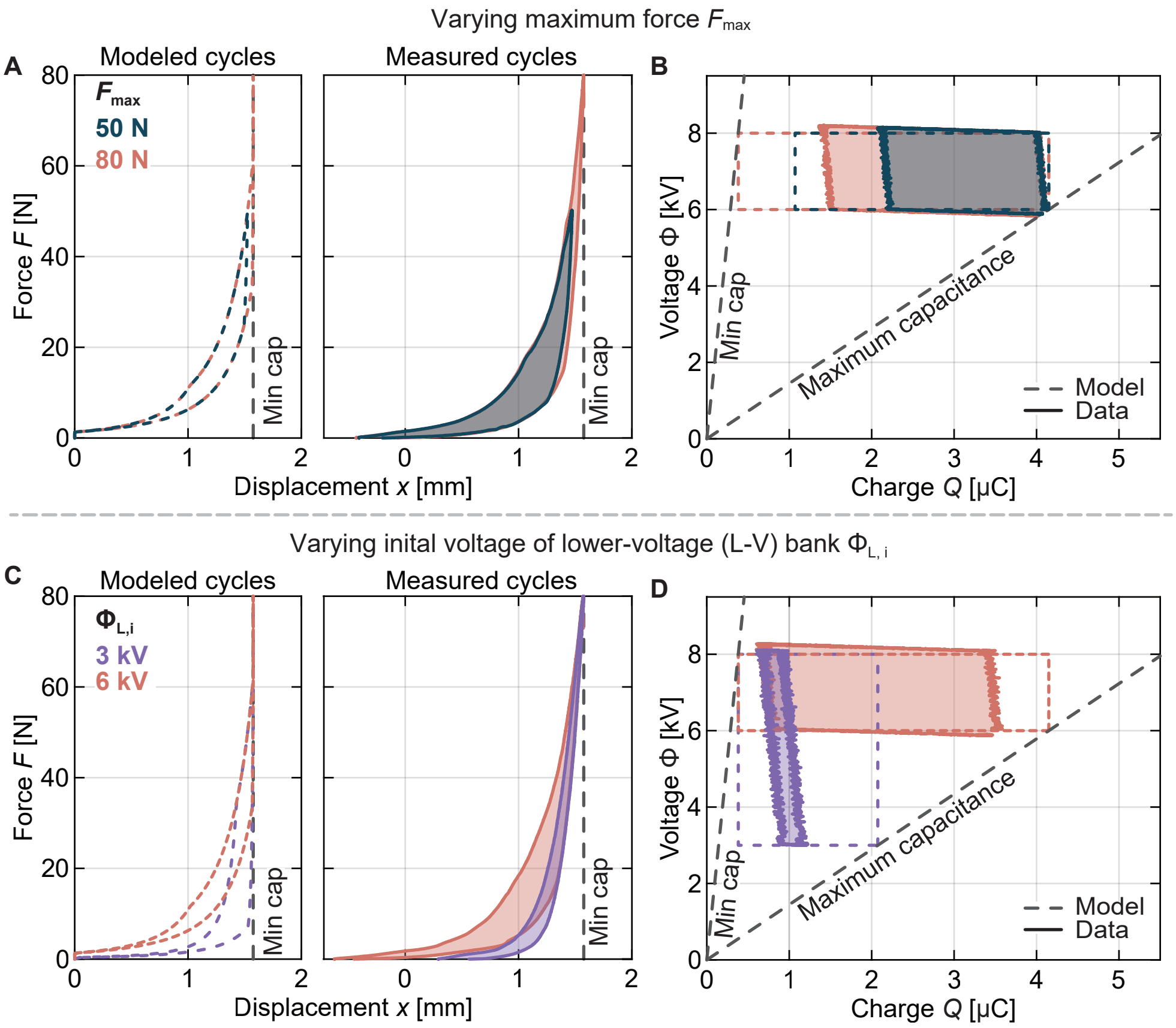

Figure S10. Model of the mechanical and electrical cycles from in Figure 5 shown on work-conjugate planes. (A, B) Varying the maximum applied force $F_{\max}$ and (C, D) varying the initial voltage of the lower-voltage bank $\Phi_{\mathrm{L,i}}$.

	Equi-biaxial DEG		Bellow transducer	Flexible ES gen	Peano-HASEL	This work		
First author	Huang [S3]	Shian [S7]	Sirbu [S8]	Yang [S10]	Hess [S2]	Kirkman		
Year	2013	2014	2021	2023	2024	2026		
Cycle shape, QV-plane	Rectangle	Triangle	Triangle, constant voltage			Rectangle		
Max. voltage [kV]	5	~4.5	6	6	6	8		
Electrode fraction	1	1	0.7	0.5	0.5	0.75		
Film thickness [μm]	500	500	25.4	25	not stated	20		
Frequency [Hz]	0.5	0.2	0.5	0.25	0.5	1	1	4
Force [N]	50-105	55	not stated	5	20	80	180	80
Mass considered	Electrode-covered elastomer		Core mass (no liquid)	Liquid + pouch + skirt	Liquid	Liquid + pouch		
Spec. energy [J/kg]	560	780	0.41	0.049	2.2*	12	16	9
Spec. power [W/kg]	280	170	0.21	0.035	1.1	12	16	36
Efficiency [%]	27	30	0.2	17.8	23**	41	30	36
Practical challenges	Very high stretch, low lifetime		Complex manufacturing	Does not zip independently	Mounts required for tension mode			

Table S1. Comparing performance metrics and operating conditions for the cited generators. Notes: *we assume that the liquid is silicone oil with a density of 0.9 $\mathrm{g/cm^3}$. **authors calculated mechanical energy input using half of a mechanical cycle ($mg\Delta x$)

.

References

[S1] G. N. Greaves, A. L. Greer, R. S. Lakes, and T. Rouxel. Poisson's ratio and modern materials. *Nature Materials*, 10:823–837, 11 2011.

[S2] Isabel Hess, Stephen Chamot, Blake Boren, and Patrick Musgrave. Generating electricity with hydraulically amplified self-healing electrostatic (HASEL) transducers. *Energies*, 17:6130, 12 2024.

[S3] Jiangshui Huang, Samuel Shian, Zhigang Suo, and David R. Clarke. Maximizing the energy density of dielectric elastomer generators using equi-biaxial loading. *Advanced Functional Materials*, 23:5056–5061, 2013.

[S4] Nicholas Kellaris, Vidyacharan Gopaluni Venkata, Philipp Rothemund, and Christoph Keplinger. An analytical model for the design of Peano-HASEL actuators with drastically improved performance. *Extreme Mechanics Letters*, 29:100449, 5 2019. check.

[S5] Shane K. Mitchell, Xingrui Wang, Eric Acome, Trent Martin, Khoi Ly, Nicholas Kellaris, Vidyacharan Gopaluni Venkata, and Christoph Keplinger. An easy-to-implement toolkit to create versatile and high-performance HASEL actuators for untethered soft robots. *Advanced Science*, 6, 7 2019.

[S6] Natalia Sanchez-Tamayo, Zachary Yoder, Philipp Rothemund, Giulia Ballardini, Christoph Keplinger, and Katherine J. Kuchenbecker. Cutaneous electrohydraulic (CUTE) wearable devices for pleasant broad-bandwidth haptic cues. *Advanced Science*, 11, 12 2024.

[S7] Samuel Shian, Jiangshui Huang, Shijie Zhu, and David R. Clarke. Optimizing the electrical energy conversion cycle of dielectric elastomer generators. *Advanced Materials*, 26:6617–6621, 10 2014.

[S8] I. D. Sîrbu, G. Moretti, G. Bortolotti, M. Bolignari, S. Diré, L. Fambri, R. Vertechy, and M. Fontana. Electrostatic bellow muscle actuators and energy harvesters that stack up. *Science Robotics*, 6:1–12, 2 2021.

[S9] S Timoshenko and S Woinowsky-Krieger. *Theory of Plates and Shells*. McGraw-Hill, 1959.

[S10] Ruisen Yang, Meng Yang, Peng Fan, Tongqing Lu, and Tiejun Wang. A new flexible electrostatic generator using dielectric fluid. *Journal of Applied Physics*, 134, 9 2023.
